# Supplementary material for: Multicentre, non-interventional study to assess the profile of patients with uncontrolled rhinitis prescribed a novel formulation of azelastine hydrochloride and fluticasone propionate in a single spray in routine clinical practice in the UK
Source: BMJ Open. 2017 Apr 24;7(4):e014777. doi: 10.1136/bmjopen-2016-014777 (PMC5775468; doi:10.1136/bmjopen-2016-014777)
Supplement: Author's manuscript [file bmjopen-2016-014777.draft_revisions.pdf]

BMJ Open

Profile of patients with uncontrolled allergic rhinitis prescribed MP-AzeFlu in routine clinical practice in the UK

Journal:	*BMJ Open*
Manuscript ID	bmjopen-2016-014777
Article Type:	Research
Date Submitted by the Author:	24-Oct-2016

Complete List of Authors:	Scadding, Glenis; Royal National TNE Hospital, AllergyRhinology<br>Price, David; University of Aberdeen; Observational and Pragmatic Research Institute Pte Ltd<br>El-Shanawany, Tariq; University Hospital of Wales<br>Ahmed, Shahzada; Queen Elizabeth Hospital Birmingham<br>Ray, Jaydip; Royal Hallamshire Hospital<br>Sargur, Ravishankar; Northern General Hospital<br>Kumar, Nirmal; Royal Albert & Edward Hospital

**Primary Subject Heading**:	Respiratory medicine
Secondary Subject Heading:	Pharmacology and therapeutics
Keywords:	allergic rhinitis, azelastine, fluticasone propionate, UK, Dymista

SCHOLARONE™ Manuscripts

Profile of patients with uncontrolled allergic rhinitis prescribed MP-AzeFlu in routine clinical practice in the UK

Glenis Scadding[1], David Price[2,3], Tariq El-Shanawany[4], Shahzada Ahmed[5], Jaydip Ray[6], Ravishankar Sargur[7] and Nirmal Kumar[8]

[1]Royal National Throat, Nose and Ear Hospital, London, UK; [2]University of Aberdeen, Aberdeen, Scotland; [3]Observational and Pragmatic Research Institute Pte Ltd, Singapore, SG; [4]Univeristy Hospital of Wales, Cardiff, UK; [5]Queen Elizabeth Hospital, Birmingham, UK; [6]Royal Hallamshire, Sheffield, UK; [7] Northern General Hospital, Sheffield, UK; [8]Royal Albert & Edward Hospital, Wigan, UK.

Address for correspondence

Glenis Scadding

University College London Hospitals NHS Foundation Trust,

Royal National Throat, Nose & Ear Hospital,

330 Gray's Inn Road, London WC1X 8DA

Tel +44 (0) 203 456 5045

Fax +44 (0) 203 456 5045

Email: g.scadding@ucl.ac.uk

Word count: 2764

Number tables: 2

For peer review only

Number of figures: 3

Number of references: 35

Key words: allergic rhinitis, azelastine, Dymista, fluticasone propionate, UK

Running head: MP-AzeFlu allergic rhinitis patient profile in the UK

For peer review only

For peer review only - http://bmjopen.bmj.com/site/about/guidelines.xhtml

ABSTRACT

Objective: The aims of this study were (i) to characterise the type of patient prescribed MP-AzeFlu (Dymista®, a novel formulation of azelastine hydrochloride, fluticasone propionate and excipients in a single spray) in real-life in the UK and physicians' reasons for prescribing it and (ii) to quantify the personal and societal burden of AR in the UK prior to MP-AzeFlu prescription.

Design, setting & participants: This multicentre, prospective, non-interventional study enrolled patients (n=193) with moderate-to-severe AR and acute symptoms who were eligible to receive treatment with MP-AzeFlu according to its licensed indications. Information was gathered on patient demographics, AR history and symptom severity, symptomatology and previous AR treatments in the last calendar year (prior to MP-AzeFlu prescription). Physicians also recorded number of previous AR visits, specific reasons for these visits and their reason for prescribing MP-AzeFlu.

Results: Most patients had seasonal AR (SAR) either alone (10.4%) or in combination with perennial AR (PAR; 35.2%), but many had AR of unknown origin (35.8%). Prior to MP-AzeFlu prescription patients reported troublesome symptoms (78.2%) and sleep disturbance (64.8%), with congestion considered the most bothersome (54.4%) and ocular symptoms reported by 68.4% of patients. The most frequent reason for MP-AzeFlu prescription was that other therapies were not sufficient in the past (78.8%) or not sufficient to treat acute symptoms (16.1%). 79.3% of patients reported using ≥2 AR therapies in the past year. An average of 1.6 (standard deviation (SD) 1.9) doctor visits due to AR were reported prior to MP-AzeFlu prescription.

For peer review only - http://bmjopen.bmj.com/site/about/guidelines.xhtml

Conclusion: many patients in the UK live with uncontrolled disease despite treatment with mono- and multiple therapies and repeat doctor visits. A more effective treatment option, like MP-AzeFlu, should improve AR control, reduce repeat doctor visits and costs associated with its management, and reduce the number of patients requiring immunotherapy.

STRENGTHS and LIMIATIONS OF THIS STUDY

- Provides important epidemiological data on the type of AR patient presenting to secondary care allergy clinics in the UK and confirms the enormous burden imposed by uncontrolled AR symptoms on patients' lives.
- Provides information on AR treatment pattern in the UK (prior to MP-AzeFlu prescription) and the shortcomings of commonly used treatments in providing symptom control.
- Explores the high socio-economic burden imposed by AR, as seen through medication usage and number of doctor visits for AR.
- Details how the indication for MP-AzeFlu, was interpreted by prescribing physicians in secondary and tertiary care during the first months of its introduction and use in the UK.
- Although this study did not access the effectiveness of MP-AzeFlu in real life, it has been shown in numerous real-life studies in other European countries.

For peer review only

For peer review only - http://bmjopen.bmj.com/site/about/guidelines.xhtml

INTRODUCTION

One in 4 individuals in the United Kingdom has allergic rhinitis (AR),[1 2] yet this disease continues to be under-estimated, both in terms of its impact on individuals and their families,[3 4] as well as its cost to the economy. When symptomatic, patients feel the impact of AR on all areas of their daily lives, at home, socially and at work/school.[5-7] Indeed, in the UK children with symptomatic AR are more likely to unexpectedly drop an exam grade during summer tests than their non-AR counterparts, and are even more at risk of so doing if taking sedating anti-histamines.[8] Poorly controlled AR also has a negative impact on asthma control, equivalent to that induced by smoking.[9] While the direct costs (e.g. drug costs) associated with AR are relatively low, the indirect costs due to absenteeism and presenteeism are disproportionately large, estimated at £1.4 billion/year in the UK alone.[10]

The burden of AR and the importance of achieving disease control has now been recognised both by the European Academy of Allergy & Clinical Immunology[11] and at the EU level.[12 13] Provision of more effective and fast acting AR treatments proven to achieve guideline-defined control in real-life should help reduce the burden of disease and associated management costs.[14]

MP-AzeFlu (Dymista®, Meda, Solna, Sweden) comprises a novel formulation containing an intranasal anti-histamine (azelastine hydrochloride), an intranasal corticosteroid (INS), fluticasone propionate (FP), and excipients delivered in a single spray. It has been approved for use in the UK since January 2013, indicated for the relief of symptoms of moderate-to-severe seasonal and perennial allergic rhinitis if monotherapy with either intranasal

For peer review only - http://bmjopen.bmj.com/site/about/guidelines.xhtml

antihistamine or glucocorticoid is not considered sufficient.[15] In a randomised controlled clinical trial (RCT) setting, MP-AzeFlu provided twice the overall nasal and ocular symptom relief as FP or azelastine mono-therapy, and provided faster complete (or near complete) relief in more patients.[16] Patients treated with MP-AzeFlu in real-life settings in Germany, Sweden, Denmark, Norway and Romania experienced rapid and sustained symptom control, with 1 in 2 patients reporting that their AR was well-controlled after 3 days.[17] In those studies, control was assessed using the a simple visual analogue scale (VAS) score, as endorsed by MACVIA-ARIA (Contre les Maladie Chronique pour un Vieillissement Actif-Allergic Rhinitis and its Impact on Asthma) and incorporated into the updated guideline, called AR clinical decision support system (CDSS).[14] On average, patients treated with MP-AzeFlu crossed the well-controlled VAS score cut off (i.e. 5/10 cm) prior to Day 3, irrespective of disease severity, phenotype (SAR, PAR, SAR + PAR and unknown) or previous treatment (with mono- or multiple therapies).[17 18]

Characteristics of patients who received MP-AzeFlu in a RCT setting are well-established, due to strict inclusion/exclusion criteria applied to ensure a homogenous patient population and low external variability.[19] However, detailed characteristics of those patients prescribed MP-AzeFlu in routine clinical practice are unknown. The aim of this study was to (i) characterise the type of patient prescribed MP-AzeFlu in real-life in the UK and physicians' reasons for prescribing it (iii) quantify the burden of AR prior to MP-AzeFlu prescription and (ii) describe AR treatment patterns for these patients prior to MP-AzeFlu use in the UK.

For peer review only - http://bmjopen.bmj.com/site/about/guidelines.xhtml

METHODS

Study design

This was a multi-centre, prospective non-interventional study conducted in the UK between October 2013 and May 2014, as part of a larger Europe-wide study of similar design. During the study, patients were treated with MP-AzeFlu (1 spray in each nostril twice-daily). The study was performed in line with current UK laws and guidelines and all study documents approved by a central Research Ethics Committee.

Patients

Patients were eligible for inclusion into the study if they were aged ≥12 years, had a documented diagnosis of moderate-to-severe seasonal AR (SAR) or perennial AR (PAR) and for whom monotherapy with either intranasal antihistamine or glucocorticoid were not considered sufficient.[15] Patients were required to have acute AR symptoms on the study inclusion day. SAR was defined as allergy to at least one pollen allergen (i.e. spring, summer and/or autumn pollen) but no non-pollen allergens. PAR was defined as allergy to at least one non-pollen allergen (i.e. dust mites, pet dander and/or mould) but no pollen allergens. SAR + PAR was defined as allergy to at least one pollen and at least one non-pollen allergen. AR of unknown origin was defined as allergy to other or unknown allergens (i.e. not one of the allergens listed above) or unknown allergens (i.e. rhinitis indicated from history but not specific IgE data).

Patients were excluded if they had hypersensitivity to azelastine hydrochloride, FP or any MP-AzeFlu excipients. There were no restrictions regarding concomitant treatments, apart

For peer review only - http://bmjopen.bmj.com/site/about/guidelines.xhtml

from ritonavir, which was to be avoided. Female patients were excluded if they were pregnant or breast feeding. All patients provided written informed consent prior to their participation in the study. If patients were below 16 years' old, their caregiver also provided written informed consent.

Physicians

Physicians who were usually involved in AR management participated in this study and included secondary care ear-nose-and-throat (ENT) specialists, immunologists and allergists. Each physician could enrol up to 20 patients. The decision whether to include a patient in the study was made by the physician independently from and after the decision to prescribe MP-AzeFlu had been made.

MP-AzeFlu use in routine clinical practice

Information on patient demographics, clinical symptoms, and previous AR treatments in the last calendar year (prior to MP-AzeFlu prescription) was documented by physicians. Physicians also recorded information on type of AR, number of visits in the current calendar year due to AR, predominant symptoms, and ARIA-defined AR severity. The reason for the patient's visit ("acute AR symptoms", "expected allergen exposure in near future" or "other") and the reason for prescribing MP-AzeFlu ("other therapies were not sufficient in the past", "other therapies are not considered to be sufficient to treat acute symptoms", or "other") was documented by the physician. All data were recorded by physicians in an English language electronic case report from (eCRF; Trium Analysis Online GmbH).

For peer review only - http://bmjopen.bmj.com/site/about/guidelines.xhtml

For peer review only

Statistics

This study included 193 AR patients, which was deemed sufficient to provide insight into use of MP-AzeFlu in routine clinical practice in the UK. Analyses were conducted on the safety population, defined as all patients who were treated at least once with MP-AzeFlu and whose physician provided an electronic signature to confirm data accuracy. All data were reported with descriptive statistics. Categorical data were described in terms of frequency and as percentage of patients. Continuous data were described as mean and standard deviation (SD). Analyses were performed by the Contract Research Organisation, Syneed Medidata GmbH, with SAS Version 9.1.3.

For peer review only - http://bmjopen.bmj.com/site/about/guidelines.xhtml

RESULTS

Patient disposition

The study was conducted in 15 study centres in the UK who enrolled 195 patients. Of these, 2 patients were excluded from data analysis due to unconfirmed data documentation, leaving 193 confirmed patients in the safety population.

Patient demographics and baseline characteristics

A summary of patient demographics and baseline clinical characteristics is presented in Table 1. There were slightly more females than males in the safety population. The mean (SD) age of the study population was 37.6 (16.9) years, with most of the patients aged 18–65 years. Almost half of patients had SAR, either alone or in combination with PAR. A relatively high proportion of patients had rhinitis with aetiology of unknown origin (Table 1). The vast majority of patients had confirmed ARIA-defined moderate-to-severe disease. AR symptoms were troublesome to patients, negatively impacting sleep, daily activities, leisure and/or sport and school/work. Nasal congestion was by far the most common predominant symptom. Ocular symptoms were present in over two-thirds of patients (Table 1).

For peer review only - http://bmjopen.bmj.com/site/about/guidelines.xhtml

Table 1. Patient demographics and baseline clinical characteristics (N=193).

Characteristic	n (%)
**Gender**	
Female	107 (55.4)
**Age, yr**	
12–17	22 (11.4)
18–65	155 (80.3)
>65	16 (8.3)
**Duration of rhinitis**,* year (mean [SD])	8.5 (9.4)
**Type of rhinitis**	
SAR	20 (10.4)
PAR	36 (18.7)
SAR + PAR	68 (35.2)
Unknown origin	69 (35.8)
**Severity of AR criteria****	
Troublesome symptoms	151 (78.2)
Sleep disturbance	125 (64.8)
Impairment of daily activities/leisure/sport	109 (56.5)

For peer review only - http://bmjopen.bmj.com/site/about/guidelines.xhtml

Impairment of school/work	93 (48.2)
At least one criteria	175 (90.7)
**Predominant symptoms**	
Nasal congestion	105 (54.4)
Rhinorrhoea	26 (13.5)
Sneezing	20 (10.4)
Nasal itching	10 (5.2)
Unknown	32 (16.6)
**Concomitant ocular symptoms**	132 (68.4)

*n=136

** Moderate/severe AR if at least one criterion was met

AR: allergic rhinitis; PAR: perennial allergic rhinitis; SAR: seasonal allergic rhinitis; SD: standard deviation.

Physician visits

In the current calendar year, the mean (SD) number of physician visits due to AR was 1.6 (1.9). 62.7% (n=121) of patients had visited their physician at least once before inclusion into the study in the current calendar year due to their AR (Figure 1); with most attending once or twice before. However, 23.8% of patients (n=46) had made three or more visits prior to the current visit (Figure 1).

For peer review only - http://bmjopen.bmj.com/site/about/guidelines.xhtml

Reasons for patient visit and MP-AzeFlu prescription

MP-AzeFlu was prescribed according to its licensed indication. The most frequent reasons for the physician visit were ‘acute AR symptoms’ (n=142; 73.6%), ‘expected allergen exposure in the near future’ (n=21; 10.9%) and ‘other’ (n=37; 19.2%). The most frequent reason for prescribing MP-AzeFlu was that ‘other therapies were not sufficient in the past’. For the remaining patients other reasons were cited, including ‘other therapies were not considered sufficient to treat acute symptoms’ (Figure 2).

AR treatments in the last year

The majority of patients had received or used at least one AR therapy, either prescription or over the counter, prior to MP-AzeFlu prescription (Table 2). The AR medications most frequently used in the last year were INS, oral antihistamines, and oral/intranasal decongestants. Eye drops, either anti-histamine or mast cell stabiliser use, was reported by over a quarter of patients. Systemic corticosteroid use was noted for quite a high proportion of patients (Table 2). The majority of patients reported using multiple AR treatments in the last year, prior to MP-AzeFlu use; dual AR therapy was most commonly reported, but many also used triple and even quadruple AR therapies (Figure 3). A total of 25 patients (13.0%) reported previously using 5 or more AR treatments prior to MP-AzeFlu prescription. Seven patients (3.6%) stated that they were undergoing immunotherapy at the time of the inclusion visit and 1 (0.5%) had received an immunotherapy course in the last calendar year.

For peer review only - http://bmjopen.bmj.com/site/about/guidelines.xhtml

Table 2. AR treatments in the past year (N=193).

AR treatments (multiple entries possible)	n (%)
Intranasal corticosteroid	162 (83.9)
Oral antihistamine	128 (66.3)
Intranasal decongestant	47 (24.4)
Oral decongestant	41 (21.2)
Systemic corticosteroid	36 (18.7)
Intranasal antihistamine	31 (16.1)
Ocular antihistamine	31 (16.1)
Ocular mast cell stabiliser	23 (11.9)
Oral leukotriene receptor antagonist	12 (6.2)
Intranasal mast cell stabiliser	1 (0.5)
Any other	11 (5.7)
Unknown	8 (4.2)
None	3 (1.6)
Immunotherapy (in past or ongoing)	8 (4.1)
Multiple therapies (excluding immunotherapy)	153 (79.3)

AR: allergic rhinitis

For peer review only - http://bmjopen.bmj.com/site/about/guidelines.xhtml

For peer review only

DISCUSSION

This study represents a valuable addition to the AR knowledge base for several reasons. Firstly, it provides important epidemiological data on the type of AR patient presenting to secondary care allergy clinics in the UK, as well as information on AR history and symptomatology of those who received MP-AzeFlu. Secondly, it confirms the enormous (and frequently under-estimated)[4] burden imposed by uncontrolled AR symptoms on patients' lives. Thirdly it provides information on AR treatment pattern in the UK (prior to MP-AzeFlu prescription) and the shortcomings of commonly used treatments in providing symptom control. It also explores the high socio-economic burden imposed by AR, as seen through medication usage and number of doctor visits for AR. Finally, this study details how the indication for MP-AzeFlu,[15] a relatively new addition to the AR armoury, was interpreted by prescribing physicians in secondary and tertiary care during the first months of its introduction and use in the UK.

Physicians who took part in this non-interventional study (i.e. allergists, ENT specialists and immunologists) prescribed MP-AzeFlu to adolescents, adults and elderly patients with moderate/severe AR (i.e. ≥1 ARIA severity criteria)[20] in line with its indication.[15] It was prescribed to those patients for whom other AR therapies were not considered sufficient, and also to those for whom other therapy had not worked in the past. This decision depends very much on physician and patient preference; i.e. the preference for rapid AR symptom control straight away with the most effective pharmacological treatment option, or a gradual therapy step up until control has been achieved. MP-AzeFlu was prescribed for the treatment of acute symptoms, and also prophylactically in the anticipation of allergen

For peer review only - http://bmjopen.bmj.com/site/about/guidelines.xhtml

exposure in the near future. It was prescribed for patients with SAR, PAR, SAR and PAR and for those whose AR aetiology was not defined, which represented about 1/3 of patients in the current study. This unknown group is likely quite heterogeneous in nature, and could include those with non-AR, or local AR, as well as those with SAR or PAR of unknown allergen or allergen not listed. Finally, MP-AzeFlu was prescribed for patients previously on AR mono-therapy and for those attending clinic for the first time in the season. It was also prescribed for patients with more complicated disease who had attended many times in the past year for AR, and for those on multiple therapies or immunotherapy.

Although MACVIA ARIA clearly recommends MP-AzeFlu for the treatment of intermittent and persistent AR,[14] a significant proportion of patients who may benefit from this treatment in the UK have not received it. The need for wider awareness and use of MP-AzeFlu in the UK (particularly in primary care) is shown by the fact that prior to MP-AzeFlu prescription the majority of patients reported troublesome symptoms, sleep disturbance and impairment of their daily activities, with almost half acknowledging a negative impact of their AR symptoms on performance at work or school. The negative impact of AR symptoms on daily activities has previously been shown to be greater for AR patients than those with diabetes mellitus or hypertension.[21] Others have shown that living with symptomatic AR can impair school performance,[8] impair driving ability to the same extent as a blood alcohol level of 0.05%,[22] reduce work productivity[10 23] and induce absenteeism.[10] The latter two carry a hefty price tag, with absenteeism due to AR estimated to cost the UK economy £1.14 billion/year[10] and the cost of lost productivity due to AR thought to exceed that of chronic heart disease, asthma, diabetes, hypertension and respiratory infections combined![23]

For peer review only - http://bmjopen.bmj.com/site/about/guidelines.xhtml

The results of this study also show that the most commonly prescribed AR treatments in the UK do not provide adequate symptom relief for many patients, further endorsing the need for MP-AzeFlu. This inadequacy was evident from the high symptomatic burden on treatment reported by patients, the high degree of polypharmacy noted (79.3%) and the high incidence of use of systemic corticosteroids (18.7%) and decongestants (45.6%). The former are rarely indicated for the management of rhinitis (except for severe obstruction or short-term rescue use),[24] whilst long-term use of the latter has been associated with rhinitis medicamentosa. A previous survey conducted in the UK also revealed that patients retained a high nasal and ocular symptom burden despite treatment with mono- or multiple AR therapies.[10] Furthermore, a retrospective observational study with data from the Optimum Patient Care Research Database showed that in the UK monotherapy with antihistamines or INS provided insufficient symptom relief for about 20% of SAR patients.[25] AR monotherapy was prescribed first for most patients, but by season end 45% of SAR patients had received multiple therapy, a practice which is not recommended by ARIA[20] due to insufficient evidence.[26 27] The current British Society for Allergy & Clinical Immunology (BSACI) UK guidelines suggest that additional therapies should be considered for brief targeted symptom relief only.[24]

The concept of achieving symptom relief (i.e. reduction in symptom severity) has been now been superseded by the concept of achieving symptom control.[14] A simple visual analogue scale (VAS) is recommended by MACVIA ARIA as the new language of AR control.[12] The VAS has been used extensively in the field of AR to assess symptom severity[28] and treatment effectiveness[29], to define phenotypes, such as severe chronic upper airway disease,[30] and to

For peer review only

define clinically relevant effect.[31] A VAS score of 5/10 cm is the cut-off used to define control and guide treatment decisions as part of an updated ARIA AR treatment algorithm called the AR CDSS.[14] It has also been incorporated into an app (*Allergy* Diary) to empower patients to take control of their own AR.[12] Data obtained from several countries (Germany, Sweden, Norway, Denmark and Romania) which gathered real-life data as part of a multi-national pan-European study have shown that on average patients treated with MP-AzeFlu achieve this AR CDSS VAS score cut-off (i.e. 5 cm) prior to Day 3, with 1 in 2 patients perceiving their AR as well-controlled at that time point.[18 32-35] The UK data presented here are taken from the same multi-national pan-European study. Although, effectiveness data were not recorded in the UK arm, results are expected to be consistent with those found in other countries with similar patient characteristics. MP-AzeFlu has the potential to reduce costs associated with managing AR in the UK, by reducing multiple prescriptions, number of doctor visits, referrals to secondary care and potentially reducing the number of patients referred for allergen-specific immunotherapy (AIT); supporting UK NHS cost-saving initiatives to manage more chronic conditions in the primary care setting.

The limitations of this study are those typically associated with non-interventional, observational datasets where there is reliance on data drawn from clinical records and from patients themselves. Another limitation is that our study included no investigation of the effectiveness of MP-AzeFlu, although this has been demonstrated in real-life studies conducted in other countries.[17]

For peer review only - http://bmjopen.bmj.com/site/about/guidelines.xhtml

In conclusion, the symptomatic burden of AR remains high for many patients in the UK, who live with uncontrolled disease, despite treatment with mono- and multiple therapies and repeat doctor visits. A more effective treatment option, like MP-AzeFlu, should improve AR control and reduce costs associated with its management.

For peer review only

For peer review only - http://bmjopen.bmj.com/site/about/guidelines.xhtml

ACKNOWLEDGEMENTS

The authors thank the following investigators for their participation in the study: Mr Carl Philpott, James Paget Hospital, Great Yarmouth; Dr Dermot Dalton North Devon District Hospital, Barnstable; Dr Sadie Khawaja, Stepping Hill Hospital, Stockport; Dr Tom Dawson, Worcester Royal Hospital, West Midlands; Mr Alagar Chandra-Mohan, Ysbyty Gwynedd Hospital, Wrexham, Wales; Mr Arul Puveendran, Sunderland Royal Hospital, Sunderland; Mr Paul Counter, Cumberland Infirmary, Carlisle; Mr Ramsam Ullah, Royal Victoria Hospital, Belfast, N.Ireland; Mr San Sunkaraneni, Royal Surrey Hospital, Guildford; Prof Chris Raine, Bradford Royal Infirmary, Bradford. We also gratefully acknowledge each of the clinical research facilities which took part in this study.

Medical writing assistance in the preparation of this manuscript was provided by Dr Ruth Murray and Dr Winnie McFadzaen, Medscript Ltd, funded by MEDA Pharma GmbH & Co. KG. We thank Dr DunTung Nguyen and Dr Hans-Christian Kuhl for critical assessment of the data and manuscript review.

Contribution of authors

GS: Was the chief investigator of the study and contributed to the study design. She also contributed to the interpretation of the data for the work, drafting, revising and critically reviewing the article for intellectual content and provided final approval to be published.

DP: Contributed to the interpretation of the data for the work, drafting, revising and critically reviewing the article for intellectual content and provided final approval to be published.

For peer review only - http://bmjopen.bmj.com/site/about/guidelines.xhtml

TE: Actively recruited patients and provided data necessary to write this article. He also contributed to the interpretation of the data for the work, drafting, revising and critically reviewing the article for intellectual content and provided final approval to be published.

SKA: Actively recruited patients and provided data necessary to write this article. He also contributed to the interpretation of the data for the work, drafting, revising and critically reviewing the article for intellectual content and provided final approval to be published.

JR: Actively recruited patients and provided data necessary to write this article. He aslo contributed to the interpretation of the data for the work, drafting, revising and critically reviewing the article for intellectual content and provided final approval to be published.

RS: Actively recruited patients and provided data necessary to write this article. He also contributed to the interpretation of the data for the work, drafting, revising and critically reviewing the article for intellectual content and provided final approval to be published.

NK: Actively recruited patients and provided data necessary to write this article. He also contributed to the interpretation of the data for the work, drafting, revising and critically reviewing the article for intellectual content and provided final approval to be published.

COMPETING INTERESTS

GS has received fees for advising Meda and for speaking at meetings on Dymista.

DP has received fees for advising Meda, research grants from Meda for studies on allergic rhinitis and fees for speaking at meetings and symposia supported by Meda

TE has received educational support, speaker fees and/or advisory board fees from Meda, Allergy Therapeutics and ALK.

SKA has received fees for speaking at a meeting by Meda.

For peer review only - http://bmjopen.bmj.com/site/about/guidelines.xhtml

For peer review only

JR has received fees for speaking at a meeting by Meda.

RS has received honorarium in MEDA sponsored educational events.

NK has received fees for advising and for speaking at meetings arranged by Meda

DATA SHARING STATEMENT

No additional data are available

FUNDING

This research was funded by Meda UK

For peer review only

For peer review only - http://bmjopen.bmj.com/site/about/guidelines.xhtml

REFERENCES

1. Bauchau V, Durham SR. Prevalence and rate of diagnosis of allergic rhinitis in Europe. *Eur Respir J* 2004;24:758-64.
2. Bauchau V, Durham SR. Epidemiological characterization of the intermittent and persistent types of allergic rhinitis. *Allergy* 2005;60:350-3.
3. Scadding GK, Williams A. The burden of allergic rhinitis as reported by UK patients compared with their doctors. *Rhinology* 2008;46:99-106.
4. Meltzer EO. Allergic rhinitis: the impact of discordant perspectives of patient and physician on treatment decisions. *Clin Ther* 2007;29:1428-40.
5. Valovirta E, Myrseth SE, Palkonen S. The voice of the patients: allergic rhinitis is not a trivial disease. *Curr Opin Allergy Clin Immunol* 2008;8:1-9.
6. Bousquet PJ, Demoly P, Devillier P, et al. Impact of allergic rhinitis symptoms on quality of life in primary care. *Int Arch Allergy Immunol* 2013;160:393-400.
7. Scadding GK, Richards DH, Price MJ. Patient and physician perspectives on the impact and management of perennial and seasonal allergic rhinitis. *Clin Otolaryngol Allied Sci* 2000;25:551-7.
8. Walker S, Khan-Wasti S, Fletcher M, et al. Seasonal allergic rhinitis is associated with a detrimental effect on examination performance in United Kingdom teenagers: case-control study. *J Allergy Clin Immunol* 2007;120:381-7.
9. Clatworthy J, Price D, Ryan D, et al. The value of self-report assessment of adherence, rhinitis and smoking in relation to asthma control. *Prim Care Respir J* 2009;18:300-5.
10. Price D, Scadding G, Ryan D, et al. The hidden burden of adult allergic rhinitis: UK healthcare resource utilisation survey. *Clin Transl Allergy* 2015;5:39.

For peer review only - http://bmjopen.bmj.com/site/about/guidelines.xhtml

11. European Academy of Allergy and Clinical Immunology (EAACI). Beware of Allergy Campaign [Available from: http://www.bewareofallergy.com accessed July 2016].

12. Bousquet J, Schunemann HJ, Fonseca J, et al. MACVIA-ARIA Sentinel NetworK for allergic rhinitis (MASK-rhinitis): the new generation guideline implementation. *Allergy* 2015;70:1372-92.

13. European Innovation Partnership on A, Healthy Ageing APB, Mechanisms of the Development of Allergy WP, et al. Integrated care pathways for airway diseases (AIRWAYS-ICPs). *Eur Respir J* 2014;44:304-23.

14. Bousquet J, Schunemann H, Arnavielhe S, et al. MACVIA clinical decision algorithm in allergic rhinitis in adolescents and adults. *J Allergy Clin Immunol* 2016;138:367-74.

15. Dymista. Summary of Product Characteristics [Available from: https://www.medicines.org.uk/emc/medicine/27579. last accessed Oct 2016]

16. Meltzer E, Ratner P, Bachert C, et al. Clinically relevant effect of a new intranasal therapy (MP29-02) in allergic rhinitis assessed by responder analysis. *Int Arch Allergy Immunol* 2013;161:369-77.

17. Klimek L, Bachert, C., Stjarne, P., Dollner, R., Larsen, P., Haahr, P., Agache, I., Scadding, G., Price, D. MP-AzeFlu provides rapid and effective allergic rhinitis control in real-life: a pan-European study. *Allergy Asthma Proc* 2016;37:376-86.

18. Stjarne P, Strand V, Theman K, et al. Real-life effectiveness of a new allergic rhinitis thrapy (MP29-02) in Sweden. *Allergy* 2015;70:504.

19. Price D, Smith P, Hellings P, et al. Current controversies and challenges in allergic rhinitis management. *Expert Rev Clin Immunol* 2015;11:1205-17.

For peer review only - http://bmjopen.bmj.com/site/about/guidelines.xhtml

20. Bousquet J, Khaltaev N, Cruz AA, et al. Allergic Rhinitis and its Impact on Asthma (ARIA) 2008 update (in collaboration with the World Health Organization, GA(2)LEN and AllerGen). *Allergy* 2008;63 Suppl 86:8-160.

21. de la Hoz Caballer B, Rodriguez M, Fraj J, et al. Allergic rhinitis and its impact on work productivity in primary care practice and a comparison with other common diseases: the Cross-sectional study to evAluate work Productivity in allergic Rhinitis compared with other common dIseases (CAPRI) study. *Am J Rhinol Allergy* 2012;26:390-4.

22. Vuurman EF, Vuurman LL, Lutgens I, et al. Allergic rhinitis is a risk factor for traffic safety. *Allergy* 2014;69:906-12.

23. Lamb CE, Ratner PH, Johnson CE, et al. Economic impact of workplace productivity losses due to allergic rhinitis compared with select medical conditions in the United States from an employer perspective. *Curr Med Res Opin* 2006;22:1203-10.

24. Scadding GK, Durham SR, Mirakian R, et al. BSACI guidelines for the management of allergic and non-allergic rhinitis. *Clin Exp Allergy* 2008;38:19-42.

25. Price DB, Scadding G, Bachert C, et al. UK prescribing practices as proxy markers of unmet need in allergic rhinitis: a retrospective observational study. *NPJ Prim Care Respir Med* 2016;26:16033.

26. Anolik R, Mometasone Furoate Nasal Spray With Loratadine Study G. Clinical benefits of combination treatment with mometasone furoate nasal spray and loratadine vs monotherapy with mometasone furoate in the treatment of seasonal allergic rhinitis. *Ann Allergy Asthma Immunol* 2008;100:264-71.

For peer review only - http://bmjopen.bmj.com/site/about/guidelines.xhtml

27. Esteitie R, deTineo M, Naclerio RM, et al. Effect of the addition of montelukast to fluticasone propionate for the treatment of perennial allergic rhinitis. *Ann Allergy Asthma Immunol* 2010;105:155-61.

28. Bousquet PJ, Combescure C, Neukirch F, et al. Visual analog scales can assess the severity of rhinitis graded according to ARIA guidelines. *Allergy* 2007;62:367-72.

29. Bousquet J, Bachert C, Canonica GW, et al. Efficacy of desloratadine in intermittent allergic rhinitis: a GA(2)LEN study. *Allergy* 2009;64:1516-23.

30. Bousquet PJ, Bachert C, Canonica GW, et al. Uncontrolled allergic rhinitis during treatment and its impact on quality of life: a cluster randomized trial. *J Allergy Clin Immunol* 2010;126:666-8 e1-5.

31. Demoly P, Bousquet PJ, Mesbah K, et al. Visual analogue scale in patients treated for allergic rhinitis: an observational prospective study in primary care: asthma and rhinitis. *Clin Exp Allergy* 2013;43:881-8.

32. Klimek L, Bachert C, Mosges R, et al. Effectiveness of MP29-02 for the treatment of allergic rhinitis in real-life: results from a noninterventional study. *Allergy Asthma Proc* 2015;36:40-7.

33. Dollner R, Lorentz Larsen P, Sheyauldeen S, et al. Real-life effectiveness of a new allergic rhinitis therapy (MP29-02) in Norway. *Allergy* 2015;70:502.

34. Haahr P, Jacobsen C, Blegvad S, et al. Real life effectiveness of a new allergic rhinitis therapy (MP29-02) in Denmark. *Allergy* 2015;70:500.

35. Agache ISCL, P; Bucur, I; Doros, I-C; Poenaru, M. Real-life effectiveness of a new allergic rhinitis therapy (MP-AzeFlu*) in Romania. *Allergy* 2016;71(Suppl S102):386.

For peer review only - http://bmjopen.bmj.com/site/about/guidelines.xhtml

LEGEND TO FIGURES

Figure 1

Number of previous physician visits for allergic rhinitis (AR) in the current calendar year prior to prescribing MP-AzeFlu. N=193 patients with moderate-to-severe AR attending routine clinical practice in the UK.

Figure 2

Most frequent reasons reported by physicians for prescribing MP-AzeFlu to patients with moderate-to-severe allergic rhinitis (n=193) attending routine clinical practice in the UK. Tx: treatment

Figure 3

Number of allergic rhinitis (AR) treatments (prescription and over the counter) used in the last calendar year by AR patients with moderate-to-severe AR (n=193) attending routine clinical practice in the UK

For peer review only

For peer review only - http://bmjopen.bmj.com/site/about/guidelines.xhtml

For peer review only

For peer review only - http://bmjopen.bmj.com/site/about/guidelines.xhtml

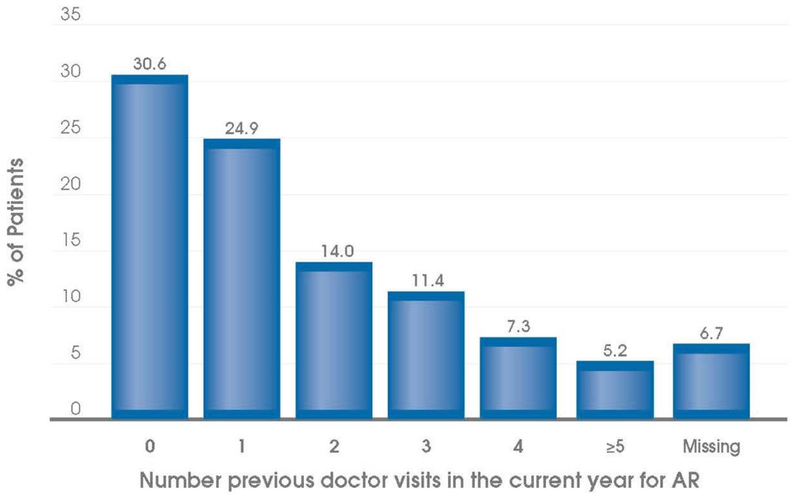

Figure 1: Number of previous physician visits for allergic rhinitis (AR) in the current calendar year prior to prescribing MP-AzeFlu. N=193 patients with moderate-to-severe AR attending routine clinical practice in the UK

200x100mm (200 x 200 DPI)

review only

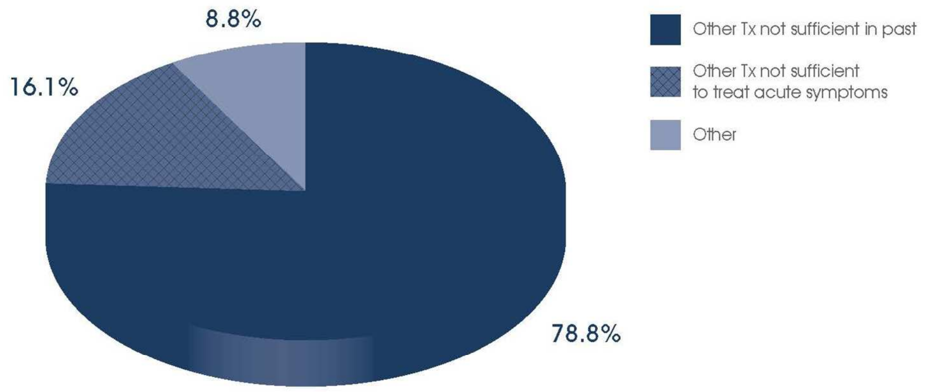

Figure 2: Most frequent reasons reported by physicians for prescribing MP-AzeFlu to patients with moderate-to-severe allergic rhinitis (n=193) attending routine clinical practice in the UK. Tx: treatment

200x100mm (200 x 200 DPI)

For peer review only

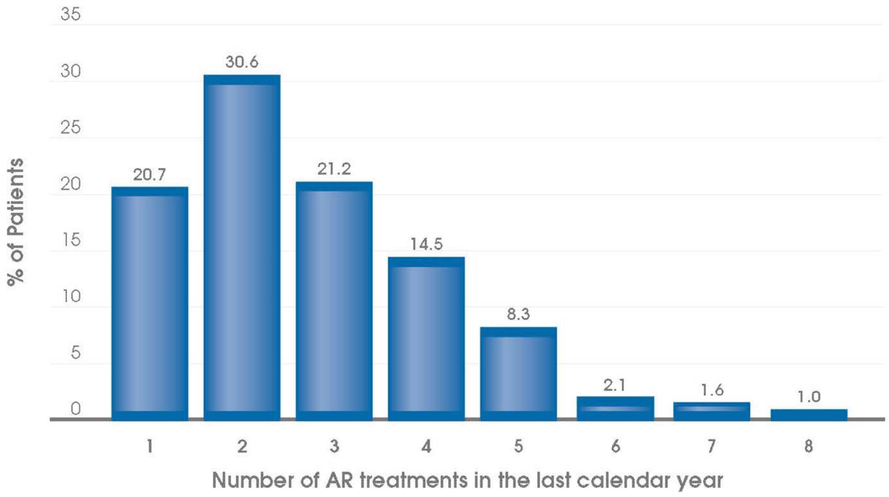

Figure 3: Number of allergic rhinitis (AR) treatments (prescription and over the counter) used in the last calendar year by AR patients with moderate-to-severe disease (n=193) attending routine clinical practice in the UK.

200x100mm (200 x 200 DPI)

review only

STROBE Statement—checklist of items that should be included in reports of observational studies

	Item No	Recommendation	
**Title and abstract**	1	(*a*) Indicate the study's design with a commonly used term in the title or the abstract	X
		(*b*) Provide in the abstract an informative and balanced summary of what was done and what was found	X
**Introduction**			
Background/rationale	2	Explain the scientific background and rationale for the investigation being reported	X
Objectives	3	State specific objectives, including any prespecified hypotheses	X
**Methods**			
Study design	4	Present key elements of study design early in the paper	X
Setting	5	Describe the setting, locations, and relevant dates, including periods of recruitment, exposure, follow-up, and data collection	X
Participants	6	(*a*) *Cohort study*—Give the eligibility criteria, and the sources and methods of selection of participants. Describe methods of follow-up<br>*Case-control study*—Give the eligibility criteria, and the sources and methods of case ascertainment and control selection. Give the rationale for the choice of cases and controls<br>*Cross-sectional study*—Give the eligibility criteria, and the sources and methods of selection of participants	X
		(*b*) *Cohort study*—For matched studies, give matching criteria and number of exposed and unexposed<br>*Case-control study*—For matched studies, give matching criteria and the number of controls per case	NA
Variables	7	Clearly define all outcomes, exposures, predictors, potential confounders, and effect modifiers. Give diagnostic criteria, if applicable	X
Data sources/ measurement	8*	For each variable of interest, give sources of data and details of methods of assessment (measurement). Describe comparability of assessment methods if there is more than one group	*X*
Bias	9	Describe any efforts to address potential sources of bias	X
Study size	10	Explain how the study size was arrived at	
Quantitative variables	11	Explain how quantitative variables were handled in the analyses. If applicable, describe which groupings were chosen and why	NA
Statistical methods	12	(*a*) Describe all statistical methods, including those used to control for confounding	NA
		(*b*) Describe any methods used to examine subgroups and interactions	NA
		(*c*) Explain how missing data were addressed	NA
		(*d*) *Cohort study*—If applicable, explain how loss to follow-up was addressed<br>*Case-control study*—If applicable, explain how matching of cases and controls was addressed<br>*Cross-sectional study*—If applicable, describe analytical methods taking account of sampling strategy	NA
		(*e*) Describe any sensitivity analyses	

Continued on next page

For peer review only

**Results**			
Participants	13*	(a) Report numbers of individuals at each stage of study—eg numbers potentially eligible, examined for eligibility, confirmed eligible, included in the study, completing follow-up, and analysed	NA
		(b) Give reasons for non-participation at each stage	NA
		(c) Consider use of a flow diagram	NA
Descriptive data	14*	(a) Give characteristics of study participants (eg demographic, clinical, social) and information on exposures and potential confounders	X
		(b) Indicate number of participants with missing data for each variable of interest	NA
		(c) *Cohort study*—Summarise follow-up time (eg, average and total amount)	NA
Outcome data	15*	*Cohort study*—Report numbers of outcome events or summary measures over time	*X*
		Case-control study—Report numbers in each exposure category, or summary measures of exposure	
		Cross-sectional study—Report numbers of outcome events or summary measures	
Main results	16	(*a*) Give unadjusted estimates and, if applicable, confounder-adjusted estimates and their precision (eg, 95% confidence interval). Make clear which confounders were adjusted for and why they were included	NA
		(*b*) Report category boundaries when continuous variables were categorized	NA
		(*c*) If relevant, consider translating estimates of relative risk into absolute risk for a meaningful time period	NA
Other analyses	17	Report other analyses done—eg analyses of subgroups and interactions, and sensitivity analyses	NA
**Discussion**			
Key results	18	Summarise key results with reference to study objectives	X
Limitations	19	Discuss limitations of the study, taking into account sources of potential bias or imprecision. Discuss both direction and magnitude of any potential bias	X
Interpretation	20	Give a cautious overall interpretation of results considering objectives, limitations, multiplicity of analyses, results from similar studies, and other relevant evidence	X
Generalisability	21	Discuss the generalisability (external validity) of the study results	X
**Other information**			
Funding	22	Give the source of funding and the role of the funders for the present study and, if applicable, for the original study on which the present article is based	X

*Give information separately for cases and controls in case-control studies and, if applicable, for exposed and unexposed groups in cohort and cross-sectional studies.

Note: An Explanation and Elaboration article discusses each checklist item and gives methodological background and published examples of transparent reporting. The STROBE checklist is best used in conjunction with this article (freely available on the Web sites of PLoS Medicine at http://www.plosmedicine.org/, Annals of Internal Medicine at http://www.annals.org/, and Epidemiology at http://www.epidem.com/). Information on the STROBE Initiative is available at www.strobe-statement.org.

For peer review only

Multi-centre, non-interventional study to assess the profile of patients with uncontrolled allergic rhinitis prescribed MP-AzeFlu in routine clinical practice in the UK

Journal:	*BMJ Open*
Manuscript ID	bmjopen-2016-014777.R1
Article Type:	Research
Date Submitted by the Author:	26-Jan-2017
Complete List of Authors:	Scadding, Glenis; Royal National TNE Hospital, AllergyRhinology<br>Price, David; University of Aberdeen; Observational and Pragmatic Research Institute Pte Ltd<br>El-Shanawany, Tariq; University Hospital of Wales<br>Ahmed, Shahzada; Queen Elizabeth Hospital Birmingham<br>Ray, Jaydip; Royal Hallamshire Hospital<br>Sargur, Ravishankar; Northern General Hospital<br>Kumar, Nirmal; Royal Albert & Edward Hospital
**Primary Subject Heading**:	Respiratory medicine
Secondary Subject Heading:	Ear, nose and throat/otolaryngology, Epidemiology
Keywords:	allergic rhinitis, azelastine, fluticasone propionate, UK, Dymista

SCHOLARONE™
Manuscripts

For peer review only - http://bmjopen.bmj.com/site/about/guidelines.xhtml

Multi-centre, non-interventional study to assess the profile of patients with uncontrolled allergic rhinitis prescribed MP-AzeFlu in routine clinical practice in the UK

Glenis Scadding[1], David Price[2,3], Tariq El-Shanawany[4], Shahzada Ahmed[5], Jaydip Ray[6], Ravishankar Sargur[7] and Nirmal Kumar[8]

[1]Royal National Throat, Nose and Ear Hospital, London, UK; [2]University of Aberdeen, Aberdeen, Scotland; [3]Observational and Pragmatic Research Institute Pte Ltd, Singapore, SG; [4]Univeristy Hospital of Wales, Cardiff, UK; [5]Queen Elizabeth Hospital, Birmingham, UK; [6]Royal Hallamshire, Sheffield, UK; [7] Northern General Hospital, Sheffield, UK; [8]Royal Albert & Edward Hospital, Wigan, UK.

Address for correspondence

Glenis Scadding

University College London Hospitals NHS Foundation Trust,

Royal National Throat, Nose & Ear Hospital,

330 Gray's Inn Road, London WC1X 8DA

Tel +44 (0) 203 456 5045

Fax +44 (0) 203 456 5045

Email: g.scadding@ucl.ac.uk

Word count: 3279

Number tables: 2

For peer review only - http://bmjopen.bmj.com/site/about/guidelines.xhtml

Number of figures: 3

Number of references: 35

Key words: allergic rhinitis, azelastine, Dymista, fluticasone propionate, UK

Running head: MP-AzeFlu allergic rhinitis patient profile in the UK

For peer review only

For peer review only - http://bmjopen.bmj.com/site/about/guidelines.xhtml

ABSTRACT

Objective: The aims of this study were (i) to characterise the type of patient prescribed MP-AzeFlu (Dymista®, a novel formulation of azelastine hydrochloride, fluticasone propionate and excipients in a single spray) in real-life in the UK and physicians' reasons for prescribing it and (ii) to quantify the personal and societal burden of AR in the UK prior to MP-AzeFlu prescription.

Design, setting & participants: This multicentre, non-interventional study enrolled patients (n=193) with moderate-to-severe AR and acute symptoms who were eligible to receive treatment with MP-AzeFlu according to its licensed indications. Information was gathered on patient demographics, AR history and symptom severity, symptomatology and previous AR treatments in the last calendar year (prior to MP-AzeFlu prescription). Physicians also recorded number of previous AR visits, specific reasons for these visits and their reason for prescribing MP-AzeFlu.

Results: Most patients had seasonal AR (SAR) either alone (10.4%) or in combination with perennial AR (PAR; 35.2%), but many had AR of unknown origin (35.8%). Prior to MP-AzeFlu prescription patients reported troublesome symptoms (78.2%) and sleep disturbance (64.8%), with congestion considered the most bothersome (54.4%) and ocular symptoms reported by 68.4% of patients. The most frequent reason for MP-AzeFlu prescription was that other therapies were not sufficient in the past (78.8%) or not sufficient to treat acute symptoms (16.1%). 79.3% of patients reported using ≥2 AR therapies in the past year. An average of 1.6 (standard deviation (SD) 1.9) doctor visits due to AR were reported prior to MP-AzeFlu prescription.

For peer review only - http://bmjopen.bmj.com/site/about/guidelines.xhtml

Conclusion: In the UK, MP-AzeFlu was prescribed for individuals (≥12 years) with moderate/severe AR irrespective of (i) previous AR treatment (mono or multiple), (ii) previous or likely treatment failure, (iii) phenotype, (iv) number of previous physician visits for AR and (v) for the relief of both acute symptoms and in anticipation of allergen exposure.

STRENGTHS and LIMIATIONS OF THIS STUDY

Strengths

- Sufficiently large patient numbers to draw general conclusion on use of MP-AzeFlu in routine clinical practice in UK.
- Low amount of missing data
- Real-life data obtained from daily practice highlighting the burden of AR in the UK and the type of patient prescribed MP-AzeFlu.
- Relates type of patient prescribed MP-AzeFlu with the labelled indication, thus providing sound estimates for relevant subgroups from a socio-economic perspective

Limitations

- For such single arm epidemiological research standard bias limiting methods (blinding, randomisation) could not be applied.
- No investigation of the effectiveness of MP-AzeFlu, although this has been demonstrated in real-life studies conducted in other countries

For peer review only - http://bmjopen.bmj.com/site/about/guidelines.xhtml

71

72

For peer review only

For peer review only - http://bmjopen.bmj.com/site/about/guidelines.xhtml

INTRODUCTION

One in 4 individuals in the United Kingdom has allergic rhinitis (AR),[1 2] yet this disease continues to be under-estimated, both in terms of its impact on individuals and their families,[3 4] as well as its cost to the economy. When symptomatic, patients feel the impact of AR on all areas of their daily lives, at home, socially and at work/school.[5-7] Indeed, in the UK children with symptomatic AR are more likely to unexpectedly drop an exam grade during summer tests than their non-AR counterparts, and are even more at risk of so doing if taking sedating anti-histamines.[8] Poorly controlled AR also has a negative impact on asthma control, equivalent to that induced by smoking.[9] While the direct costs (e.g. drug costs) associated with AR are relatively low, the indirect costs due to absenteeism and presenteeism are disproportionately large, estimated at £1.4 billion/year in the UK alone.[10]

The burden of AR and the importance of achieving disease control has now been recognised both by the European Academy of Allergy & Clinical Immunology[11] and at the EU level.[12 13] Provision of more effective and fast acting AR treatments proven to achieve guideline-defined control in real-life should help reduce the burden of disease and associated management costs.[14]

MP-AzeFlu (Dymista®, Meda, Solna, Sweden) comprises a novel formulation containing an intranasal anti-histamine (azelastine hydrochloride), an intranasal corticosteroid (INS), fluticasone propionate (FP), and excipients delivered in a single spray. It has been approved for use in the UK since January 2013, indicated for the relief of symptoms of moderate-to-severe seasonal and perennial allergic rhinitis if monotherapy with either intranasal

For peer review only - http://bmjopen.bmj.com/site/about/guidelines.xhtml

antihistamine or glucocorticoid is not considered sufficient.[15] In a randomised controlled clinical trial (RCT) setting, MP-AzeFlu provided twice the overall nasal and ocular symptom relief as FP or azelastine mono-therapy, and provided faster complete (or near complete) relief in more patients.[16] Patients treated with MP-AzeFlu in real-life settings in Germany, Sweden, Denmark, Norway and Romania experienced rapid and sustained symptom control, with 1 in 2 patients reporting that their AR was well-controlled after 3 days.[17] In those studies, control was assessed using the a simple visual analogue scale (VAS) score, as endorsed by MACVIA-ARIA (Contre les Maladie Chronique pour un Vieillissement Actif-Allergic Rhinitis and its Impact on Asthma) and incorporated into the updated guideline, called AR clinical decision support system (CDSS).[14] On average, patients treated with MP-AzeFlu crossed the well-controlled VAS score cut off (i.e. 5/10 cm) prior to Day 3, irrespective of disease severity, phenotype (SAR, PAR, SAR + PAR and unknown) or previous treatment (with mono- or multiple therapies).[17]

Characteristics of patients who received MP-AzeFlu in a RCT setting are well-established, due to strict inclusion/exclusion criteria applied to ensure a homogenous patient population and low external variability.[18] However, detailed characteristics of those patients prescribed MP-AzeFlu in routine clinical practice are unknown. The aim of this study was to (i) characterise the type of patient prescribed MP-AzeFlu in real-life in the UK and physicians' reasons for prescribing it (iii) quantify the burden of AR prior to MP-AzeFlu prescription and (ii) describe AR treatment patterns for these patients prior to MP-AzeFlu use in the UK. The effectiveness of MP-AzeFlu in a real-life pan-European clinical setting has already been established.[17]

For peer review only - http://bmjopen.bmj.com/site/about/guidelines.xhtml

METHODS

Study design

This was a multi-centre, non-interventional study conducted in the UK between October 2013 and May 2014, as part of a larger Europe-wide study of similar design. During the study, patients were treated with MP-AzeFlu (1 spray in each nostril twice-daily). MP-AzeFlu is indicated for the relief of symptoms of moderate to severe seasonal and perennial allergic rhinitis if monotherapy with either intranasal antihistamine or glucocorticoid is not considered sufficient.[15] The study was performed in line with current UK laws and guidelines and all study documents approved by a central Research Ethics Committee (National Research Ethics Service Committee London –Brent). The study was registered at the European Network of Centres for Pharmacoepidemiology and Pharmacovigilance.

Patients

Patients were eligible for inclusion into the study if they were aged ≥12 years, had a documented diagnosis of moderate-to-severe seasonal AR (SAR) or perennial AR (PAR) and for whom monotherapy with either intranasal antihistamine or glucocorticoid were not considered sufficient.[15] Patients were required to have acute AR symptoms on the study inclusion day. SAR patients were defined as those with a documented allergy to at least one pollen allergen (i.e. spring, summer and/or autumn pollen) but no non-pollen allergens. PAR patients were those with a documented allergy to at least one non-pollen allergen (i.e. dust mites, pet dander and/or mould) but no pollen allergens. Patients with both SAR + PAR had a documented allergy to at least one pollen and at least one non-pollen allergen. Rhinits of unknown origin was defined as allergy to other or unknown allergens (i.e. not one of the

For peer review only - http://bmjopen.bmj.com/site/about/guidelines.xhtml

For peer review only

allergens listed above) or unknown allergens (i.e. rhinitis indicated from history but no specific IgE data)

Patients were excluded if they had hypersensitivity to azelastine hydrochloride, FP or any MP-AzeFlu excipients. There were no restrictions regarding concomitant treatments, apart from ritonavir, which was to be avoided. Female patients were excluded if they were pregnant or breast feeding. All patients provided written informed consent prior to their participation in the study. If patients were below 16 years' old, their caregiver also provided written informed consent.

Physicians

Physicians who were usually involved in AR management participated in this study and included secondary care ear-nose-and-throat (ENT) specialists, immunologists and allergists. These physicians were identified by an independent agency (pH Associated, UK). Each physician could enrol up to 20 patients. The decision whether to include a patient in the study was made by the physician independently from and after the decision to prescribe MP-AzeFlu had been made.

MP-AzeFlu use in routine clinical practice

Information on patient demographics, clinical symptoms, and previous AR treatments in the last calendar year (prior to MP-AzeFlu prescription) was documented by physicians. Physicians also recorded information on type of AR, number of visits in the current calendar

For peer review only - http://bmjopen.bmj.com/site/about/guidelines.xhtml

year due to AR, predominant symptoms, and ARIA-defined AR severity. The reason for the patient's visit ("acute AR symptoms", "expected allergen exposure in near future" or "other") and the reason for prescribing MP-AzeFlu ("other therapies were not sufficient in the past", "other therapies are not considered to be sufficient to treat acute symptoms", or "other") was documented by the physician. All data were recorded by physicians in a pre-specified English language electronic case report from (eCRF; Trium Analysis Online GmbH) based on the investigators' patient files and on information obtained at the patient visit.

Statistics

This study included 193 AR patients, which was deemed sufficient to provide insight into use of MP-AzeFlu in routine clinical practice in the UK. Analyses were conducted on the safety population, defined as all patients who were treated at least once with MP-AzeFlu and whose physician provided an electronic signature to confirm data accuracy. All data were reported with descriptive statistics. Categorical data were described in terms of frequency and as percentage of patients. Continuous data were described as mean and standard deviation (SD). Analyses were performed by the Contract Research Organisation, Syneed Medidata GmbH, with SAS Version 9.1.3.

For peer review only - http://bmjopen.bmj.com/site/about/guidelines.xhtml

RESULTS

Patient disposition

The study was conducted in 15 study centres in the UK who enrolled 195 patients. Of these, 2 patients were excluded from data analysis due to unconfirmed data documentation, leaving 193 confirmed patients in the safety population.

Patient demographics and baseline characteristics

A summary of patient demographics and baseline clinical characteristics is presented in Table 1. There were slightly more females than males in the safety population. The mean (SD) age of the study population was 37.6 (16.9) years, with most of the patients aged 18–65 years. Almost half of patients had SAR, either alone or in combination with PAR. A relatively high proportion of patients had rhinitis with aetiology of unknown origin (Table 1). The vast majority of patients had confirmed ARIA-defined moderate-to-severe disease. AR symptoms were troublesome to patients, negatively impacting sleep, daily activities, leisure and/or sport and school/work. Nasal congestion was by far the most common predominant symptom. Ocular symptoms were present in over two-thirds of patients (Table 1).

For peer review only - http://bmjopen.bmj.com/site/about/guidelines.xhtml

197 **Table 1.** Patient demographics and baseline clinical characteristics (N=193).

Characteristic	n (%)
**Gender**	
Female	107 (55.4)
**Age, years**	
12–17	22 (11.4)
18–65	155 (80.3)
>65	16 (8.3)
**Duration of rhinitis**,* year (mean [SD])	8.5 (9.4)
**Type of rhinitis**	
SAR	20 (10.4)
PAR	36 (18.6)
SAR + PAR	68 (35.2)
Unknown origin	69 (35.8)
**Severity of AR criteria****	
Troublesome symptoms	151 (78.2)
Sleep disturbance	125 (64.8)
Impairment of daily activities/leisure/sport	109 (56.5)

For peer review only - http://bmjopen.bmj.com/site/about/guidelines.xhtml

Impairment of school/work	93 (48.2)
At least one criteria	175 (90.7)
**Predominant symptoms**	
Nasal congestion	105 (54.4)
Rhinorrhoea	26 (13.5)
Sneezing	20 (10.4)
Nasal itching	10 (5.2)
Unknown	32 (16.6)
**Concomitant ocular symptoms**	132 (68.4)

198 *n=136

199 ** Moderate/severe AR if at least one criterion was met

200 AR: allergic rhinitis; PAR: perennial allergic rhinitis; SAR: seasonal allergic rhinitis; SD:
201 standard deviation.

202

203 **Physician visits**

204 In the current calendar year, the mean (SD) number of physician visits due to AR was 1.6 (1.9). 62.7% (n=121) of patients had visited their physician at least once before inclusion into the study in the current calendar year due to their AR (Figure 1); with most attending once or twice before. However, 23.8% of patients (n=46) had made three or more visits prior to the current visit (Figure 1).

For peer review only - http://bmjopen.bmj.com/site/about/guidelines.xhtml

Reasons for patient visit and MP-AzeFlu prescription

MP-AzeFlu was prescribed according to its licensed indication. The most frequent reasons for the physician visit were 'acute AR symptoms' (n=142; 73.6%), 'expected allergen exposure in the near future' (n=21; 10.9%) and 'other' (n=37; 19.2%). The most frequent reason for prescribing MP-AzeFlu was that 'other therapies were not sufficient in the past'. For the remaining patients other reasons were cited, including 'other therapies were not considered sufficient to treat acute symptoms' (Figure 2).

AR treatments in the last year

The majority of patients had received or used at least one AR therapy, either prescription or over the counter, prior to MP-AzeFlu prescription (Table 2). The AR medications most frequently used in the last year were INS, oral antihistamines, and oral/intranasal decongestants. Eye drops, either anti-histamine or mast cell stabiliser use, was reported by over a quarter of patients. Systemic corticosteroid use was noted for quite a high proportion of patients (Table 2). The majority of patients reported using multiple AR treatments in the last year, prior to MP-AzeFlu use; dual AR therapy was most commonly reported, but many also used triple and even quadruple AR therapies (Figure 3). A total of 25 patients (13.0%) reported previously using 5 or more AR treatments prior to MP-AzeFlu prescription. Seven patients (3.6%) stated that they were undergoing immunotherapy at the time of the inclusion visit and 1 (0.5%) had received an immunotherapy course in the last calendar year.

For peer review only - http://bmjopen.bmj.com/site/about/guidelines.xhtml

230 **Table 2.** AR treatments in the past year (N=193).

AR treatments (multiple entries possible)	n (%)
Intranasal corticosteroid	162 (83.9)
Oral antihistamine	128 (66.3)
Intranasal decongestant	47 (24.4)
Oral decongestant	41 (21.2)
Systemic corticosteroid	36 (18.7)
Intranasal antihistamine	31 (16.1)
Ocular antihistamine	31 (16.1)
Ocular mast cell stabiliser	23 (11.9)
Oral leukotriene receptor antagonist	12 (6.2)
Intranasal mast cell stabiliser	1 (0.5)
Any other	11 (5.7)
Unknown	8 (4.2)
None	3 (1.6)
Immunotherapy (in past or ongoing)	8 (4.1)
Multiple therapies (excluding immunotherapy)	153 (79.3)

231 AR: allergic rhinitis

232

For peer review only

For peer review only - http://bmjopen.bmj.com/site/about/guidelines.xhtml

DISCUSSION

This study represents a valuable addition to the AR knowledge base for several reasons. Firstly, it provides important epidemiological data on the type of AR patient presenting to secondary care allergy clinics in the UK, as well as information on AR history and symptomatology of those who received MP-AzeFlu. Secondly, it confirms the enormous (and frequently under-estimated)[4] burden imposed by uncontrolled AR symptoms on patients' lives. Thirdly it provides information on AR treatment pattern in the UK (prior to MP-AzeFlu prescription) and the shortcomings of commonly used treatments in providing symptom control. It also explores the high socio-economic burden imposed by AR, as seen through medication usage and number of doctor visits for AR. Finally, this study details how the indication for MP-AzeFlu,[15] a relatively new addition to the AR armoury, was interpreted by prescribing physicians in secondary and tertiary care during the first months of its introduction and use in the UK.

Physicians who took part in this non-interventional study (i.e. allergists, ENT specialists and immunologists) prescribed MP-AzeFlu to adolescents, adults and elderly patients with moderate/severe AR (i.e. ≥1 ARIA severity criteria)[19] in line with its indication.[15] It was prescribed to those patients for whom other AR therapies were not considered sufficient, and also to those for whom other therapy had not worked in the past. This decision depends very much on physician and patient preference; i.e. the preference for rapid AR symptom control straight away with the most effective pharmacological treatment option, or a gradual therapy step up until control has been achieved. MP-AzeFlu was prescribed for the treatment of acute symptoms, and also prophylactically in the anticipation of allergen

For peer review only - http://bmjopen.bmj.com/site/about/guidelines.xhtml

exposure in the near future. It was prescribed for patients with SAR, PAR, SAR and PAR and for those whose AR aetiology was not defined, which represented about 1/3 of patients in the current study. This unknown group is likely quite heterogeneous in nature, and could include those with non-AR, or local AR, as well as those with SAR or PAR of unknown allergen or allergen not listed. Finally, MP-AzeFlu was prescribed for patients previously on AR mono-therapy and for those attending clinic for the first time in the season. It was also prescribed for patients with more complicated disease who had attended many times in the past year for AR, and for those on multiple therapies or immunotherapy.

Although MACVIA ARIA clearly recommends MP-AzeFlu for the treatment of intermittent and persistent AR,[14] a significant proportion of patients who may benefit from this treatment in the UK have not received it. The need for wider awareness and use of MP-AzeFlu in the UK (particularly in primary care) is shown by the fact that prior to MP-AzeFlu prescription the majority of patients reported troublesome symptoms, sleep disturbance and impairment of their daily activities, with almost half acknowledging a negative impact of their AR symptoms on performance at work or school. The negative impact of AR symptoms on daily activities has previously been shown to be greater for AR patients than those with diabetes mellitus or hypertension.[20] Others have shown that living with symptomatic AR can impair school performance,[8] impair driving ability to the same extent as a blood alcohol level of 0.05%,[21] reduce work productivity[10 22] and induce absenteeism.[10] The latter two carry a hefty price tag, with absenteeism due to AR estimated to cost the UK economy £1.14 billion/year[10] and the cost of lost productivity due to AR thought to exceed that of chronic heart disease, asthma, diabetes, hypertension and respiratory infections combined![22]

For peer review only - http://bmjopen.bmj.com/site/about/guidelines.xhtml

The results of this study also show that the most commonly prescribed AR treatments in the UK do not provide adequate symptom relief for many patients, further endorsing the need for MP-AzeFlu. Prescribing physicians considered that other therapies were not sufficient in the past for 66.4% of patients and would not provide adequate symptom relief for 34.5% of patients. Most patients prescribed MP-AzeFlu had previously been treated with INS and/or oral anti-histamines. Inadequacy of previous AR treatments was also evident from the high symptomatic burden on treatment reported by patients, the high degree of polypharmacy noted (79.3%) and the high incidence of use of systemic corticosteroids (18.7%) and decongestants (45.6%). The former are rarely indicated for the management of rhinitis (except for severe obstruction or short-term rescue use),[23] whilst long-term use of the latter has been associated with rhinitis medicamentosa. A previous survey conducted in the UK also revealed that patients retained a high nasal and ocular symptom burden despite treatment with mono- or multiple AR therapies.[10] Furthermore, a retrospective observational study with data from the Optimum Patient Care Research Database showed that in the UK monotherapy with antihistamines or INS provided insufficient symptom relief for about 20% of SAR patients.[24] AR monotherapy was prescribed first for most patients, but by season end 45% of SAR patients had received multiple therapy, a practice which is not recommended by ARIA[19] due to insufficient evidence.[25 26] The current British Society for Allergy & Clinical Immunology (BSACI) UK guidelines suggest that additional therapies should be considered for brief targeted symptom relief only.[23]

The concept of achieving symptom relief (i.e. reduction in symptom severity) has been now been superseded by the concept of achieving symptom control.[14] A simple visual analogue

For peer review only
For peer review only - http://bmjopen.bmj.com/site/about/guidelines.xhtml

scale (VAS) is recommended by MACVIA ARIA as the new language of AR control.[12] The VAS has been used extensively in the field of AR to assess symptom severity[27] and treatment effectiveness[28], to define phenotypes, such as severe chronic upper airway disease,[29] and to define clinically relevant effect.[30] A VAS score of 5/10 cm is the cut-off used to define control and guide treatment decisions as part of an updated ARIA AR treatment algorithm called the AR CDSS.[14] It has also been incorporated into an app (*Allergy* Diary) to empower patients to take control of their own AR.[12] Data obtained from several countries (Germany, Sweden, Norway, Denmark and Romania) which gathered real-life data as part of a multi-national pan-European study have shown that on average patients treated with MP-AzeFlu achieve this AR CDSS VAS score cut-off (i.e. 5 cm) prior to Day 3, with 1 in 2 patients perceiving their AR as well-controlled at that time point.[31-35] The UK data presented here are taken from the same multi-national pan-European study. Although, effectiveness data were not recorded in the UK arm, results are expected to be consistent with those found in other countries with similar patient characteristics. MP-AzeFlu has the potential to reduce costs associated with managing AR in the UK, by reducing multiple prescriptions, number of doctor visits, referrals to secondary care and potentially reducing the number of patients referred for allergen-specific immunotherapy (AIT); supporting UK NHS cost-saving initiatives to manage more chronic conditions in the primary care setting.

The limitations of this study are those typically associated with non-interventional, observational datasets as the standard methods of blinding and randomisation cannot be applied. Thus, confounding can never safely be ruled out. . Another limitation is that our study included no investigation of the effectiveness of MP-AzeFlu, although this has been

For peer review only - http://bmjopen.bmj.com/site/about/guidelines.xhtml

demonstrated in real-life studies conducted in other countries.[17] The strength of the evidence is derived from the inclusion of a sufficiently large patient population to draw general conclusion on use and safety of MP-AzeFlu in routine clinical practice in UK. The type of patient prescribed MP-AzeFlu in the UK was consistent with the patient profile identified in other countries including Germany, Sweden, Denmark and Romania, supporting the generalizability of our data to a wider pan-European population. In the pooled analysis for all countries as well as for the presented UK data the study data were complete for most demographic variables, therefore missing data are not considered as a potential source of bias for the study results.

In conclusion, the symptomatic burden of AR remains high for many patients in the UK, who live with uncontrolled disease, despite treatment with mono- and multiple therapies and repeat doctor visits. In the UK, MP-AzeFlu was prescribed for individuals (≥12 years) with moderate/severe AR irrespective of (i) previous AR treatment (mono or multiple), (ii) previous or likely treatment failure, (iii) phenotype, (iv) number of previous physician visits for AR and (v) for the relief of both acute symptoms and in anticipation of allergen exposure. A more effective treatment option, like MP-AzeFlu, with established superiority over intranasal corticosteroid and anti-histamine monotherapy, should improve AR control.

For peer review only - http://bmjopen.bmj.com/site/about/guidelines.xhtml

ACKNOWLEDGEMENTS

The authors thank the following investigators for their participation in the study: Mr Carl Philpott, James Paget Hospital, Great Yarmouth; Dr Dermot Dalton North Devon District Hospital, Barnstable; Dr Sadie Khawaja, Stepping Hill Hospital, Stockport; Dr Tom Dawson, Worcester Royal Hospital, West Midlands; Mr Alagar Chandra-Mohan, Ysbyty Gwynedd Hospital, Wrexham, Wales; Mr Arul Puveendran, Sunderland Royal Hospital, Sunderland; Mr Paul Counter, Cumberland Infirmary, Carlisle; Mr Ramsam Ullah, Royal Victoria Hospital, Belfast, N.Ireland; Mr San Sunkaraneni, Royal Surrey Hospital, Guildford; Prof Chris Raine, Bradford Royal Infirmary, Bradford. We also gratefully acknowledge each of the clinical research facilities which took part in this study.

Medical writing assistance in the preparation of this manuscript was provided by Dr Ruth Murray and Dr Winnie McFadzaen, Medscript Ltd, funded by MEDA Pharma GmbH & Co. KG. We thank Dr DunTung Nguyen and Dr Hans-Christian Kuhl for critical assessment of the data and manuscript review.

Contribution of authors

All authors meet the authorship standards as outlined by the International Committee of Medical Journal Editors. They have all substantially contributed to the analysis and interpretation of data contained in this study, have been integrally involved in drafting and revising the article and have provided final approval for publication. All authors have agreed to be accountable for all aspects of the work in ensuring that questions related to the accuracy or integrity of any part of this work are appropriately investigated and resolved.

For peer review only - http://bmjopen.bmj.com/site/about/guidelines.xhtml

367 **COMPETING INTERESTS**

368 GS has received fees for advising Meda and for speaking at meetings on Dymista.

369 DP has received fees for advising Meda, research grants from Meda for studies on allergic
370 rhinitis and fees for speaking at meetings and symposia supported by Meda

371 TE has received educational support, speaker fees and/or advisory board fees from Meda,
372 Allergy Therapeutics and ALK.

373 SKA has received fees for speaking at a meeting by Meda.

374 JR has received fees for speaking at a meeting by Meda.

375 RS has received honorarium in MEDA sponsored educational events.

376 NK has received fees for advising and for speaking at meetings arranged by Meda

377

For peer review only

For peer review only - http://bmjopen.bmj.com/site/about/guidelines.xhtml

REFERENCES

1. Bauchau V, Durham SR. Prevalence and rate of diagnosis of allergic rhinitis in Europe. *Eur Respir J* 2004;24(5):758-64. doi: 10.1183/09031936.04.00013904

2. Bauchau V, Durham SR. Epidemiological characterization of the intermittent and persistent types of allergic rhinitis. *Allergy* 2005;60(3):350-3. doi: 10.1111/j.1398-9995.2005.00751.x

3. Scadding GK, Williams A. The burden of allergic rhinitis as reported by UK patients compared with their doctors. *Rhinology* 2008;46(2):99-106.

4. Meltzer EO. Allergic rhinitis: the impact of discordant perspectives of patient and physician on treatment decisions. *Clin Ther* 2007;29(7):1428-40. doi: 10.1016/j.clinthera.2007.07.013

5. Valovirta E, Myrseth SE, Palkonen S. The voice of the patients: allergic rhinitis is not a trivial disease. *Curr Opin Allergy Clin Immunol* 2008;8(1):1-9. doi: 10.1097/ACI.0b013e3282f3f42f

6. Bousquet PJ, Demoly P, Devillier P, et al. Impact of allergic rhinitis symptoms on quality of life in primary care. *Int Arch Allergy Immunol* 2013;160(4):393-400. doi: 10.1159/000342991

7. Scadding GK, Richards DH, Price MJ. Patient and physician perspectives on the impact and management of perennial and seasonal allergic rhinitis. *Clin Otolaryngol Allied Sci* 2000;25(6):551-7.

8. Walker S, Khan-Wasti S, Fletcher M, et al. Seasonal allergic rhinitis is associated with a detrimental effect on examination performance in United Kingdom teenagers: case-

For peer review only - http://bmjopen.bmj.com/site/about/guidelines.xhtml

control study. *J Allergy Clin Immunol* 2007;120(2):381-7. doi: 10.1016/j.jaci.2007.03.034

9. Clatworthy J, Price D, Ryan D, et al. The value of self-report assessment of adherence, rhinitis and smoking in relation to asthma control. *Prim Care Respir J* 2009;18(4):300-5. doi: 10.4104/pcrj.2009.00037

10. Price D, Scadding G, Ryan D, et al. The hidden burden of adult allergic rhinitis: UK healthcare resource utilisation survey. *Clin Transl Allergy* 2015;5:39. doi: 10.1186/s13601-015-0083-6

11. European Academy of Allergy and Clinical Immunology (EAACI). Beware of Allergy Campaign [Available from: http://www.bewareofallergy.com accessed July 2016.

12. Bousquet J, Schunemann HJ, Fonseca J, et al. MACVIA-ARIA Sentinel NetworK for allergic rhinitis (MASK-rhinitis): the new generation guideline implementation. *Allergy* 2015;70(11):1372-92. doi: 10.1111/all.12686

13. European Innovation Partnership on A, Healthy Ageing APB, Mechanisms of the Development of Allergy WP, et al. Integrated care pathways for airway diseases (AIRWAYS-ICPs). *Eur Respir J* 2014;44(2):304-23. doi: 10.1183/09031936.00014614

14. Bousquet J, Schunemann H, Arnavielhe S, et al. MACVIA clinical decision algorithm in allergic rhinitis in adolescents and adults. *J Allergy Clin Immunol* 2016;138:367-74.

15. Dymista. Summary of Product Characteristics [Available from: https://www.medicines.org.uk/emc/medicine/27579.

16. Meltzer E, Ratner P, Bachert C, et al. Clinically relevant effect of a new intranasal therapy (MP29-02) in allergic rhinitis assessed by responder analysis. *Int Arch Allergy Immunol* 2013;161(4):369-77. doi: 10.1159/000351404

For peer review only

For peer review only - http://bmjopen.bmj.com/site/about/guidelines.xhtml

17. Klimek L, Bachert, C., Stjarne, P., Dollner, R., Larsen, P., Haahr, P., Agache, I., Scadding, G., Price, D. MP-AzeFlu provides rapid and effective allergic rhinitis control in real-life: a pan-European study. *Allergy Asthma Proc* 2016;37(5):376.

18. Price D, Smith P, Hellings P, et al. Current controversies and challenges in allergic rhinitis management. *Expert Rev Clin Immunol* 2015;11(11):1205-17. doi: 10.1586/1744666X.2015.1081814

19. Bousquet J, Khaltaev N, Cruz AA, et al. Allergic Rhinitis and its Impact on Asthma (ARIA) 2008 update (in collaboration with the World Health Organization, GA(2)LEN and AllerGen). *Allergy* 2008;63 Suppl 86:8-160. doi: 10.1111/j.1398-9995.2007.01620.x

20. de la Hoz Caballer B, Rodriguez M, Fraj J, et al. Allergic rhinitis and its impact on work productivity in primary care practice and a comparison with other common diseases: the Cross-sectional study to evAluate work Productivity in allergic Rhinitis compared with other common dIseases (CAPRI) study. *Am J Rhinol Allergy* 2012;26(5):390-4. doi: 10.2500/ajra.2012.26.3799

21. Vuurman EF, Vuurman LL, Lutgens I, et al. Allergic rhinitis is a risk factor for traffic safety. *Allergy* 2014;69(7):906-12. doi: 10.1111/all.12418

22. Lamb CE, Ratner PH, Johnson CE, et al. Economic impact of workplace productivity losses due to allergic rhinitis compared with select medical conditions in the United States from an employer perspective. *Curr Med Res Opin* 2006;22(6):1203-10. doi: 10.1185/030079906X112552

23. Scadding GK, Durham SR, Mirakian R, et al. BSACI guidelines for the management of allergic and non-allergic rhinitis. *Clin Exp Allergy* 2008;38(1):19-42. doi: 10.1111/j.1365-2222.2007.02888.x

For peer review only - http://bmjopen.bmj.com/site/about/guidelines.xhtml

24. Price DB, Scadding G, Bachert C, et al. UK prescribing practices as proxy markers of unmet need in allergic rhinitis: a retrospective observational study. *NPJ Prim Care Respir Med* 2016;26:16033. doi: 10.1038/npjpcrm.2016.33

25. Anolik R, Mometasone Furoate Nasal Spray With Loratadine Study G. Clinical benefits of combination treatment with mometasone furoate nasal spray and loratadine vs monotherapy with mometasone furoate in the treatment of seasonal allergic rhinitis. *Ann Allergy Asthma Immunol* 2008;100(3):264-71. doi: 10.1016/S1081-1206(10)60452-8

26. Esteitie R, deTineo M, Naclerio RM, et al. Effect of the addition of montelukast to fluticasone propionate for the treatment of perennial allergic rhinitis. *Ann Allergy Asthma Immunol* 2010;105(2):155-61. doi: 10.1016/j.anai.2010.05.017

27. Bousquet PJ, Combescure C, Neukirch F, et al. Visual analog scales can assess the severity of rhinitis graded according to ARIA guidelines. *Allergy* 2007;62(4):367-72. doi: 10.1111/j.1398-9995.2006.01276.x

28. Bousquet J, Bachert C, Canonica GW, et al. Efficacy of desloratadine in intermittent allergic rhinitis: a GA(2)LEN study. *Allergy* 2009;64(10):1516-23. doi: 10.1111/j.1398-9995.2009.02115.x

29. Bousquet PJ, Bachert C, Canonica GW, et al. Uncontrolled allergic rhinitis during treatment and its impact on quality of life: a cluster randomized trial. *J Allergy Clin Immunol* 2010;126(3):666-8 e1-5. doi: 10.1016/j.jaci.2010.06.034

30. Demoly P, Bousquet PJ, Mesbah K, et al. Visual analogue scale in patients treated for allergic rhinitis: an observational prospective study in primary care: asthma and rhinitis. *Clin Exp Allergy* 2013;43(8):881-8. doi: 10.1111/cea.12121

For peer review only - http://bmjopen.bmj.com/site/about/guidelines.xhtml

31. Klimek L, Bachert C, Mosges R, et al. Effectiveness of MP29-02 for the treatment of allergic rhinitis in real-life: results from a noninterventional study. *Allergy Asthma Proc* 2015;36(1):40-7. doi: 10.2500/aap.2015.36.3823

32. Stjarne P, Strand V, Theman K, et al. Real-life effectiveness of a new allergic rhinitis thrapy (MP29-02) in Sweden. *Allergy* 2015;70(504)

33. Dollner R, Lorentz Larsen P, Sheyauldeen S, et al. Real-life effectiveness of a new allergic rhinitis therapy (MP29-02) in Norway. *Allergy* 2015;70(502)

34. Haahr P, Jacobsen C, Blegvad S, et al. Real life effectiveness of a new allergic rhinitis therapy (MP29-02) in Denmark. *Allergy* 2015;70(500)

35. Agache ISCL, P; Bucur, I; Doros, I-C; Poenaru, M. Real-life effectiveness of a new allergic rhinitis therapy (MP-AzeFlu*) in Romania. *Allergy* 2016;71(Suppl S102):386.

For peer review only - http://bmjopen.bmj.com/site/about/guidelines.xhtml

481 **LEGEND TO FIGURES**

482 **Figure 1**

483 Number of previous physician visits for allergic rhinitis (AR) in the current calendar year
484 prior to prescribing MP-AzeFlu. N=193 patients with moderate-to-severe AR attending
485 routine clinical practice in the UK.

486 **Figure 2**

487 Most frequent reasons reported by physicians for prescribing MP-AzeFlu to patients with
488 moderate-to-severe allergic rhinitis (n=193) attending routine clinical practice in the UK. Tx:
489 treatment

490 **Figure 3**

491 Number of allergic rhinitis (AR) treatments (prescription and over the counter) used in the
492 last calendar year by AR patients with moderate-to-severe AR (n=193) attending routine
493 clinical practice in the UK

For peer review only - http://bmjopen.bmj.com/site/about/guidelines.xhtml

For peer review only

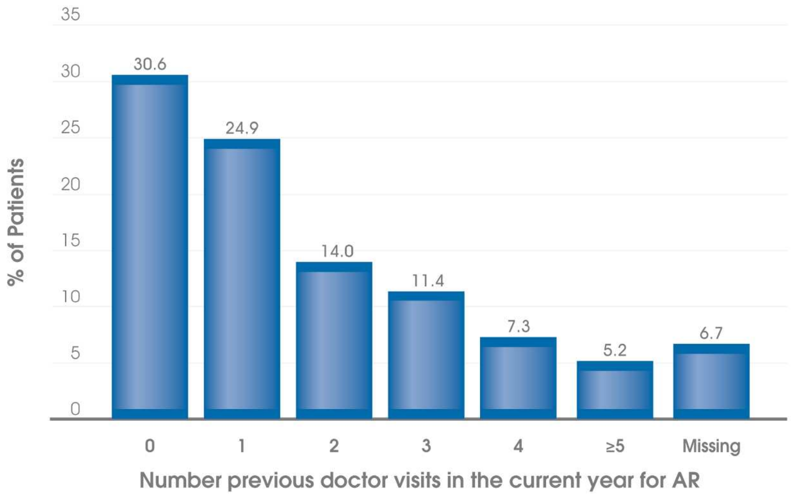

Number of previous physician visits for allergic rhinitis (AR) in the current calendar year prior to prescribing MP-AzeFlu. N=193 patients with moderate-to-severe AR attending routine clinical practice in the UK.

200x100mm (300 x 300 DPI)

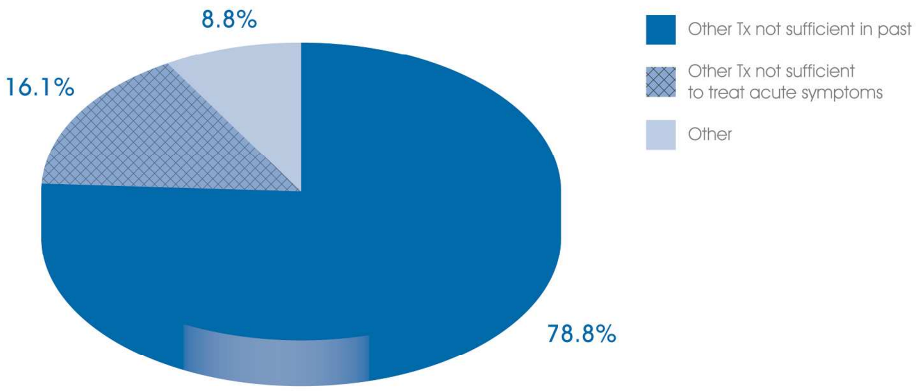

Most frequent reasons reported by physicians for prescribing MP-AzeFlu to patients with moderate-to-severe allergic rhinitis (n=193) attending routine clinical practice in the UK. Tx: treatment

200x100mm (300 x 300 DPI)

For peer review only

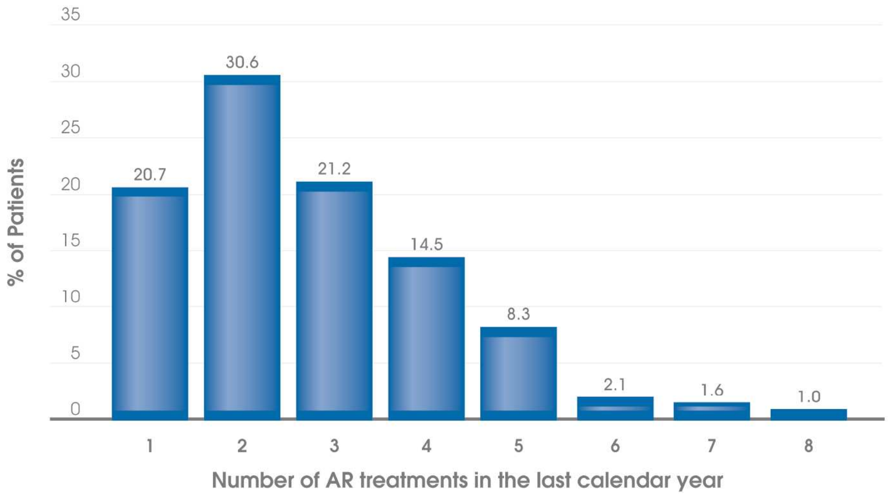

Number of allergic rhinitis (AR) treatments (prescription and over the counter) used in the last calendar year by AR patients with moderate-to-severe AR (n=193) attending routine clinical practice in the UK

200x100mm (300 x 300 DPI)

For peer review only

STROBE Statement—checklist of items that should be included in reports of observational studies

	Item No	Recommendation		Page
**Title and abstract**	1	(*a*) Indicate the study's design with a commonly used term in the title or the abstract	X	1
		(*b*) Provide in the abstract an informative and balanced summary of what was done and what was found	X	2-3
**Introduction**				
Background/rationale	2	Explain the scientific background and rationale for the investigation being reported	X	5-6
Objectives	3	State specific objectives, including any prespecified hypotheses	X	6
**Methods**				
Study design	4	Present key elements of study design early in the paper	X	7
Setting	5	Describe the setting, locations, and relevant dates, including periods of recruitment, exposure, follow-up, and data collection	X	7-8
Participants	6	(*a*) *Cohort study*—Give the eligibility criteria, and the sources and methods of selection of participants. Describe methods of follow-up<br>*Case-control study*—Give the eligibility criteria, and the sources and methods of case ascertainment and control selection. Give the rationale for the choice of cases and controls<br>*Cross-sectional study*—Give the eligibility criteria, and the sources and methods of selection of participants	X	7-8
		(*b*) *Cohort study*—For matched studies, give matching criteria and number of exposed and unexposed<br>*Case-control study*—For matched studies, give matching criteria and the number of controls per case	NA	
Variables	7	Clearly define all outcomes, exposures, predictors, potential confounders, and effect modifiers. Give diagnostic criteria, if applicable	X	8-9
Data sources/ measurement	8*	For each variable of interest, give sources of data and details of methods of assessment (measurement). Describe comparability of assessment methods if there is more than one group	*X*	*9*
Bias	9	Describe any efforts to address potential sources of bias	X	3
Study size	10	Explain how the study size was arrived at	X	9
Quantitative variables	11	Explain how quantitative variables were handled in the analyses. If applicable, describe which groupings were chosen and why	NA	
Statistical methods	12	(*a*) Describe all statistical methods, including those used to control for confounding	X	9
		(*b*) Describe any methods used to examine subgroups and interactions	NA	
		(*c*) Explain how missing data were addressed	X	19
		(*d*) *Cohort study*—If applicable, explain how loss to follow-up was addressed<br>*Case-control study*—If applicable, explain how matching of cases and controls was addressed<br>*Cross-sectional study*—If applicable, describe analytical methods taking account of sampling strategy	NA	
		(*e*) Describe any sensitivity analyses		

Continued on next page

For peer review only - http://bmjopen.bmj.com/site/about/guidelines.xhtml

**Results**				
Participants	13*	(a) Report numbers of individuals at each stage of study—eg numbers potentially eligible, examined for eligibility, confirmed eligible, included in the study, completing follow-up, and analysed	X	10
		(b) Give reasons for non-participation at each stage	X	10
		(c) Consider use of a flow diagram	NA	
Descriptive data	14*	(a) Give characteristics of study participants (eg demographic, clinical, social) and information on exposures and potential confounders	X	10-12
		(b) Indicate number of participants with missing data for each variable of interest	NA	
		(c) *Cohort study*—Summarise follow-up time (eg, average and total amount)	NA	
Outcome data	15*	*Cohort study*—Report numbers of outcome events or summary measures over time	X	*12-14*
		Case-control study—Report numbers in each exposure category, or summary measures of exposure	NA	
		Cross-sectional study—Report numbers of outcome events or summary measures	NA	
Main results	16	(*a*) Give unadjusted estimates and, if applicable, confounder-adjusted estimates and their precision (eg, 95% confidence interval). Make clear which confounders were adjusted for and why they were included	NA	
		(*b*) Report category boundaries when continuous variables were categorized	NA	
		(*c*) If relevant, consider translating estimates of relative risk into absolute risk for a meaningful time period	NA	
Other analyses	17	Report other analyses done—eg analyses of subgroups and interactions, and sensitivity analyses	NA	
**Discussion**				
Key results	18	Summarise key results with reference to study objectives	X	15 & 19
Limitations	19	Discuss limitations of the study, taking into account sources of potential bias or imprecision. Discuss both direction and magnitude of any potential bias	X	18
Interpretation	20	Give a cautious overall interpretation of results considering objectives, limitations, multiplicity of analyses, results from similar studies, and other relevant evidence	X	19
Generalisability	21	Discuss the generalisability (external validity) of the study results	X	19
**Other information**				
Funding	22	Give the source of funding and the role of the funders for the present study and, if applicable, for the original study on which the present article is based		20

*Give information separately for cases and controls in case-control studies and, if applicable, for exposed and unexposed groups in cohort and cross-sectional studies.

Note: An Explanation and Elaboration article discusses each checklist item and gives methodological background and published examples of transparent reporting. The STROBE checklist is best used in conjunction with this article (freely available on the Web sites of PLoS Medicine at http://www.plosmedicine.org/, Annals of Internal Medicine at http://www.annals.org/, and Epidemiology at http://www.epidem.com/). Information on the STROBE Initiative is available at www.strobe-statement.org.

For peer review only

BMJ Open

Multi-centre, non-interventional study to assess the profile of patients with uncontrolled rhinitis prescribed a novel formulation of azelastine hydrochloride and fluticasone propionate in a single spray in routine clinical practice in the UK

For peer review only

Journal:	*BMJ Open*
Manuscript ID	bmjopen-2016-014777.R2
Article Type:	Research
Date Submitted by the Author:	15-Feb-2017
Complete List of Authors:	Scadding, Glenis; Royal National TNE Hospital, AllergyRhinology<br>Price, David; University of Aberdeen; Observational and Pragmatic Research Institute Pte Ltd<br>El-Shanawany, Tariq; University Hospital of Wales<br>Ahmed, Shahzada; Queen Elizabeth Hospital Birmingham<br>Ray, Jaydip; Royal Hallamshire Hospital<br>Sargur, Ravishankar; Northern General Hospital<br>Kumar, Nirmal; Royal Albert & Edward Hospital
**Primary Subject Heading**:	Respiratory medicine
Secondary Subject Heading:	Ear, nose and throat/otolaryngology, Epidemiology
Keywords:	allergic rhinitis, azelastine, fluticasone propionate, UK, Dymista

SCHOLARONE™
Manuscripts

Multi-centre, non-interventional study to assess the profile of patients with uncontrolled rhinitis prescribed a novel formulation of azelastine hydrochloride and fluticasone propionate in a single spray in routine clinical practice in the UK

Glenis Scadding[1], David Price[2,3], Tariq El-Shanawany[4], Shahzada Ahmed[5], Jaydip Ray[6], Ravishankar Sargur[7] and Nirmal Kumar[8]

[1]Royal National Throat, Nose and Ear Hospital, London, UK; [2]University of Aberdeen, Aberdeen, Scotland; [3]Observational and Pragmatic Research Institute Pte Ltd, Singapore, SG; [4]Univeristy Hospital of Wales, Cardiff, UK; [5]Queen Elizabeth Hospital, Birmingham, UK; [6]Royal Hallamshire, Sheffield, UK; [7] Northern General Hospital, Sheffield, UK; [8]Royal Albert & Edward Hospital, Wigan, UK.

Address for correspondence

Glenis Scadding

University College London Hospitals NHS Foundation Trust,

Royal National Throat, Nose & Ear Hospital,

330 Gray's Inn Road, London WC1X 8DA

Tel +44 (0) 203 456 5045

Fax +44 (0) 203 456 5045

Email: g.scadding@ucl.ac.uk

Word count: 3279

Number tables: 2

Number of figures: 3

For peer review only

For peer review only - http://bmjopen.bmj.com/site/about/guidelines.xhtml

Number of references: 35

Key words: allergic rhinitis, azelastine, Dymista, fluticasone propionate, UK

Running head: MP-AzeFlu allergic rhinitis patient profile in the UK

For peer review only

For peer review only - http://bmjopen.bmj.com/site/about/guidelines.xhtml

ABSTRACT

Objective: The aims of this study were (i) to characterise the type of patient prescribed MP-AzeFlu (Dymista®, a novel formulation of azelastine hydrochloride, fluticasone propionate and excipients in a single spray) in real-life in the UK and physicians' reasons for prescribing it and (ii) to quantify the personal and societal burden of AR in the UK prior to MP-AzeFlu prescription.

Design, setting & participants: This multicentre, non-interventional study enrolled patients (n=193) with moderate-to-severe AR and acute symptoms who were eligible to receive treatment with MP-AzeFlu according to its licensed indications. Information was gathered on patient demographics, AR history and symptom severity, symptomatology and previous AR treatments in the last calendar year (prior to MP-AzeFlu prescription). Physicians also recorded number of previous AR visits, specific reasons for these visits and their reason for prescribing MP-AzeFlu.

Results: Most patients had seasonal AR (SAR) either alone (10.4%) or in combination with perennial AR (PAR; 35.2%), but many had AR of unknown origin (35.8%). Prior to MP-AzeFlu prescription patients reported troublesome symptoms (78.2%) and sleep disturbance (64.8%), with congestion considered the most bothersome (54.4%) and ocular symptoms reported by 68.4% of patients. The most frequent reason for MP-AzeFlu prescription was that other therapies were not sufficient in the past (78.8%) or not sufficient to treat acute symptoms (16.1%). 79.3% of patients reported using ≥2 AR therapies in the past year. An average of 1.6 (standard deviation (SD) 1.9) doctor visits due to AR were reported prior to MP-AzeFlu prescription.

For peer review only - http://bmjopen.bmj.com/site/about/guidelines.xhtml

Conclusion: In the UK, MP-AzeFlu was prescribed for individuals (≥12 years) with moderate/severe AR irrespective of (i) previous AR treatment (mono or multiple), (ii) previous or likely treatment failure, (iii) phenotype, (iv) number of previous physician visits for AR and (v) for the relief of both acute symptoms and in anticipation of allergen exposure.

STRENGTHS and LIMIATIONS OF THIS STUDY

Strengths

- Sufficiently large patient numbers to draw general conclusion on use of MP-AzeFlu in routine clinical practice in UK.
- Low amount of missing data
- Real-life data obtained from daily practice highlighting the burden of AR in the UK and the type of patient prescribed MP-AzeFlu.
- Relates type of patient prescribed MP-AzeFlu with the labelled indication, thus providing sound estimates for relevant subgroups from a socio-economic perspective

Limitations

- For such single arm epidemiological research standard bias limiting methods (blinding, randomisation) could not be applied.
- No investigation of the effectiveness of MP-AzeFlu, although this has been demonstrated in real-life studies conducted in other countries

For peer review only - http://bmjopen.bmj.com/site/about/guidelines.xhtml

For peer review only

For peer review only - http://bmjopen.bmj.com/site/about/guidelines.xhtml

INTRODUCTION

One in 4 individuals in the United Kingdom has allergic rhinitis (AR),[1 2] yet this disease continues to be under-estimated, both in terms of its impact on individuals and their families,[3 4] as well as its cost to the economy. When symptomatic, patients feel the impact of AR on all areas of their daily lives, at home, socially and at work/school.[5-7] Indeed, in the UK children with symptomatic AR are more likely to unexpectedly drop an exam grade during summer tests than their non-AR counterparts, and are even more at risk of so doing if taking sedating anti-histamines.[8] Poorly controlled AR also has a negative impact on asthma control, equivalent to that induced by smoking.[9] While the direct costs (e.g. drug costs) associated with AR are relatively low, the indirect costs due to absenteeism and presenteeism are disproportionately large, estimated at £1.4 billion/year in the UK alone.[10]

The burden of AR and the importance of achieving disease control has now been recognised both by the European Academy of Allergy & Clinical Immunology[11] and at the EU level.[12 13] Provision of more effective and fast acting AR treatments proven to achieve guideline-defined control in real-life should help reduce the burden of disease and associated management costs.[14]

MP-AzeFlu (Dymista®, Meda, Solna, Sweden) comprises a novel formulation containing an intranasal anti-histamine (azelastine hydrochloride), an intranasal corticosteroid (INS), fluticasone propionate (FP), and excipients delivered in a single spray. It has been approved for use in the UK since January 2013, indicated for the relief of symptoms of moderate-to-severe seasonal and perennial allergic rhinitis if monotherapy with either intranasal

For peer review only - http://bmjopen.bmj.com/site/about/guidelines.xhtml

For peer review only

antihistamine or glucocorticoid is not considered sufficient.[15] In a randomised controlled clinical trial (RCT) setting, MP-AzeFlu provided twice the overall nasal and ocular symptom relief as FP or azelastine mono-therapy, and provided faster complete (or near complete) relief in more patients.[16] Patients treated with MP-AzeFlu in real-life settings in Germany, Sweden, Denmark, Norway and Romania experienced rapid and sustained symptom control, with 1 in 2 patients reporting that their AR was well-controlled after 3 days.[17] In those studies, control was assessed using the a simple visual analogue scale (VAS) score, as endorsed by MACVIA-ARIA (Contre les Maladie Chronique pour un Vieillissement Actif-Allergic Rhinitis and its Impact on Asthma) and incorporated into the updated guideline, called AR clinical decision support system (CDSS).[14] On average, patients treated with MP-AzeFlu crossed the well-controlled VAS score cut off (i.e. 5/10 cm) prior to Day 3, irrespective of disease severity, phenotype (SAR, PAR, SAR + PAR and unknown) or previous treatment (with mono- or multiple therapies).[17]

Characteristics of patients who received MP-AzeFlu in a RCT setting are well-established, due to strict inclusion/exclusion criteria applied to ensure a homogenous patient population and low external variability.[18] However, detailed characteristics of those patients prescribed MP-AzeFlu in routine clinical practice are unknown. The aim of this study was to (i) characterise the type of patient prescribed MP-AzeFlu in real-life in the UK and physicians' reasons for prescribing it (iii) quantify the burden of AR prior to MP-AzeFlu prescription and (ii) describe AR treatment patterns for these patients prior to MP-AzeFlu use in the UK. The effectiveness of MP-AzeFlu in a real-life pan-European clinical setting has already been established.[17]

For peer review only - http://bmjopen.bmj.com/site/about/guidelines.xhtml

METHODS

Study design

This was a multi-centre, non-interventional study conducted in the UK between October 2013 and May 2014, as part of a larger Europe-wide study of similar design. During the study, patients were treated with MP-AzeFlu (1 spray in each nostril twice-daily). MP-AzeFlu is indicated for the relief of symptoms of moderate to severe seasonal and perennial allergic rhinitis if monotherapy with either intranasal antihistamine or glucocorticoid is not considered sufficient.[15] The study was performed in line with current UK laws and guidelines and all study documents approved by a central Research Ethics Committee (National Research Ethics Service Committee London –Brent). The study was registered at the European Network of Centres for Pharmacoepidemiology and Pharmacovigilance.

Patients

Patients were eligible for inclusion into the study if they were aged ≥12 years, had a documented diagnosis of moderate-to-severe seasonal AR (SAR) or perennial AR (PAR) and for whom monotherapy with either intranasal antihistamine or glucocorticoid were not considered sufficient.[15] Patients were required to have acute AR symptoms on the study inclusion day. SAR patients were defined as those with a documented allergy to at least one pollen allergen (i.e. spring, summer and/or autumn pollen) but no non-pollen allergens. PAR patients were those with a documented allergy to at least one non-pollen allergen (i.e. dust mites, pet dander and/or mould) but no pollen allergens. Patients with both SAR + PAR had a documented allergy to at least one pollen and at least one non-pollen allergen. Rhinitis of unknown origin was defined as allergy to other or unknown allergens (i.e. not one of the

For peer review only - http://bmjopen.bmj.com/site/about/guidelines.xhtml

allergens listed above) or unknown allergens (i.e. rhinitis indicated from history but no specific IgE data)

Patients were excluded if they had hypersensitivity to azelastine hydrochloride, FP or any MP-AzeFlu excipients. There were no restrictions regarding concomitant treatments, apart from ritonavir, which was to be avoided. Female patients were excluded if they were pregnant or breast feeding. All patients provided written informed consent prior to their participation in the study. If patients were below 16 years' old, their caregiver also provided written informed consent.

Physicians

Physicians who were usually involved in AR management participated in this study and included secondary care ear-nose-and-throat (ENT) specialists, immunologists and allergists. These physicians were identified by an independent agency (pH Associated, UK). Each physician could enrol up to 20 patients. The decision whether to include a patient in the study was made by the physician independently from and after the decision to prescribe MP-AzeFlu had been made.

MP-AzeFlu use in routine clinical practice

Information on patient demographics, clinical symptoms, and previous AR treatments in the last calendar year (prior to MP-AzeFlu prescription) was documented by physicians. Physicians also recorded information on type of AR, number of visits in the current calendar

For peer review only - http://bmjopen.bmj.com/site/about/guidelines.xhtml

year due to AR, predominant symptoms, and ARIA-defined AR severity. The reason for the patient's visit ("acute AR symptoms", "expected allergen exposure in near future" or "other") and the reason for prescribing MP-AzeFlu ("other therapies were not sufficient in the past", "other therapies are not considered to be sufficient to treat acute symptoms", or "other") was documented by the physician. All data were recorded by physicians in a pre-specified English language electronic case report from (eCRF; Trium Analysis Online GmbH) based on the investigators' patient files and on information obtained at the patient visit.

Statistics

This study included 193 AR patients, which was deemed sufficient to provide insight into use of MP-AzeFlu in routine clinical practice in the UK. Analyses were conducted on the safety population, defined as all patients who were treated at least once with MP-AzeFlu and whose physician provided an electronic signature to confirm data accuracy. All data were reported with descriptive statistics. Categorical data were described in terms of frequency and as percentage of patients. Continuous data were described as mean and standard deviation (SD). Analyses were performed by the Contract Research Organisation, Syneed Medidata GmbH, with SAS Version 9.1.3.

For peer review only

For peer review only - http://bmjopen.bmj.com/site/about/guidelines.xhtml

RESULTS

Patient disposition

The study was conducted in 15 study centres in the UK who enrolled 195 patients. Of these, 2 patients were excluded from data analysis due to unconfirmed data documentation, leaving 193 confirmed patients in the safety population.

Patient demographics and baseline characteristics

A summary of patient demographics and baseline clinical characteristics is presented in Table 1. There were slightly more females than males in the safety population. The mean (SD) age of the study population was 37.6 (16.9) years, with most of the patients aged 18–65 years. Almost half of patients had SAR, either alone or in combination with PAR. A relatively high proportion of patients had rhinitis with aetiology of unknown origin (Table 1). The vast majority of patients had confirmed ARIA-defined moderate-to-severe disease. AR symptoms were troublesome to patients, negatively impacting sleep, daily activities, leisure and/or sport and school/work. Nasal congestion was by far the most common predominant symptom. Ocular symptoms were present in over two-thirds of patients (Table 1).

For peer review only - http://bmjopen.bmj.com/site/about/guidelines.xhtml

197 **Table 1.** Patient demographics and baseline clinical characteristics (N=193).

Characteristic	n (%)
**Gender**	
Female	107 (55.4)
**Age, years**	
12–17	22 (11.4)
18–65	155 (80.3)
>65	16 (8.3)
**Duration of rhinitis**,* year (mean [SD])	8.5 (9.4)
**Type of rhinitis**	
SAR	20 (10.4)
PAR	36 (18.6)
SAR + PAR	68 (35.2)
Unknown origin	69 (35.8)
**Severity of AR criteria****	
Troublesome symptoms	151 (78.2)
Sleep disturbance	125 (64.8)
Impairment of daily activities/leisure/sport	109 (56.5)

For peer review only - http://bmjopen.bmj.com/site/about/guidelines.xhtml

Impairment of school/work	93 (48.2)
At least one criteria	175 (90.7)
**Predominant symptoms**	
Nasal congestion	105 (54.4)
Rhinorrhoea	26 (13.5)
Sneezing	20 (10.4)
Nasal itching	10 (5.2)
Unknown	32 (16.6)
**Concomitant ocular symptoms**	132 (68.4)

198 *n=136

199 ** Moderate/severe AR if at least one criterion was met

200 AR: allergic rhinitis; PAR: perennial allergic rhinitis; SAR: seasonal allergic rhinitis; SD:
201 standard deviation.

202

203 **Physician visits**

204 In the current calendar year, the mean (SD) number of physician visits due to AR was 1.6
205 (1.9). 62.7% (n=121) of patients had visited their physician at least once before inclusion
206 into the study in the current calendar year due to their AR (Figure 1); with most attending
207 once or twice before. However, 23.8% of patients (n=46) had made three or more visits
208 prior to the current visit (Figure 1).

For peer review only - http://bmjopen.bmj.com/site/about/guidelines.xhtml

Reasons for patient visit and MP-AzeFlu prescription

MP-AzeFlu was prescribed according to its licensed indication. The most frequent reasons for the physician visit were 'acute AR symptoms' (n=142; 73.6%), 'expected allergen exposure in the near future' (n=21; 10.9%) and 'other' (n=37; 19.2%). The most frequent reason for prescribing MP-AzeFlu was that 'other therapies were not sufficient in the past'. For the remaining patients other reasons were cited, including 'other therapies were not considered sufficient to treat acute symptoms' (Figure 2).

AR treatments in the last year

The majority of patients had received or used at least one AR therapy, either prescription or over the counter, prior to MP-AzeFlu prescription (Table 2). The AR medications most frequently used in the last year were INS, oral antihistamines, and oral/intranasal decongestants. Eye drops, either anti-histamine or mast cell stabiliser use, was reported by over a quarter of patients. Systemic corticosteroid use was noted for quite a high proportion of patients (Table 2). The majority of patients reported using multiple AR treatments in the last year, prior to MP-AzeFlu use; dual AR therapy was most commonly reported, but many also used triple and even quadruple AR therapies (Figure 3). A total of 25 patients (13.0%) reported previously using 5 or more AR treatments prior to MP-AzeFlu prescription. Seven patients (3.6%) stated that they were undergoing immunotherapy at the time of the inclusion visit and 1 (0.5%) had received an immunotherapy course in the last calendar year.

For peer review only - http://bmjopen.bmj.com/site/about/guidelines.xhtml

230 **Table 2.** AR treatments in the past year (N=193).

AR treatments (multiple entries possible)	n (%)
Intranasal corticosteroid	162 (83.9)
Oral antihistamine	128 (66.3)
Intranasal decongestant	47 (24.4)
Oral decongestant	41 (21.2)
Systemic corticosteroid	36 (18.7)
Intranasal antihistamine	31 (16.1)
Ocular antihistamine	31 (16.1)
Ocular mast cell stabiliser	23 (11.9)
Oral leukotriene receptor antagonist	12 (6.2)
Intranasal mast cell stabiliser	1 (0.5)
Any other	11 (5.7)
Unknown	8 (4.2)
None	3 (1.6)
Immunotherapy (in past or ongoing)	8 (4.1)
Multiple therapies (excluding immunotherapy)	153 (79.3)

231 AR: allergic rhinitis

232

For peer review only

For peer review only - http://bmjopen.bmj.com/site/about/guidelines.xhtml

DISCUSSION

This study represents a valuable addition to the AR knowledge base for several reasons. Firstly, it provides important epidemiological data on the type of AR patient presenting to secondary care allergy clinics in the UK, as well as information on AR history and symptomatology of those who received MP-AzeFlu. Secondly, it confirms the enormous (and frequently under-estimated)[4] burden imposed by uncontrolled AR symptoms on patients' lives. Thirdly it provides information on AR treatment pattern in the UK (prior to MP-AzeFlu prescription) and the shortcomings of commonly used treatments in providing symptom control. It also explores the high socio-economic burden imposed by AR, as seen through medication usage and number of doctor visits for AR. Finally, this study details how the indication for MP-AzeFlu,[15] a relatively new addition to the AR armoury, was interpreted by prescribing physicians in secondary and tertiary care during the first months of its introduction and use in the UK.

Physicians who took part in this non-interventional study (i.e. allergists, ENT specialists and immunologists) prescribed MP-AzeFlu to adolescents, adults and elderly patients with moderate/severe AR (i.e. ≥1 ARIA severity criteria)[19] in line with its indication.[15] It was prescribed to those patients for whom other AR therapies were not considered sufficient, and also to those for whom other therapy had not worked in the past. This decision depends very much on physician and patient preference; i.e. the preference for rapid AR symptom control straight away with the most effective pharmacological treatment option, or a gradual therapy step up until control has been achieved. MP-AzeFlu was prescribed for the treatment of acute symptoms, and also prophylactically in the anticipation of allergen

For peer review only - http://bmjopen.bmj.com/site/about/guidelines.xhtml

exposure in the near future. It was prescribed for patients with SAR, PAR, SAR and PAR and for those whose AR aetiology was not defined, which represented about 1/3 of patients in the current study. This unknown group is likely quite heterogeneous in nature, and could include those with non-AR, or local AR, as well as those with SAR or PAR of unknown allergen or allergen not listed. Finally, MP-AzeFlu was prescribed for patients previously on AR mono-therapy and for those attending clinic for the first time in the season. It was also prescribed for patients with more complicated disease who had attended many times in the past year for AR, and for those on multiple therapies or immunotherapy.

Although MACVIA ARIA clearly recommends MP-AzeFlu for the treatment of intermittent and persistent AR,[14] a significant proportion of patients who may benefit from this treatment in the UK have not received it. The need for wider awareness and use of MP-AzeFlu in the UK (particularly in primary care) is shown by the fact that prior to MP-AzeFlu prescription the majority of patients reported troublesome symptoms, sleep disturbance and impairment of their daily activities, with almost half acknowledging a negative impact of their AR symptoms on performance at work or school. The negative impact of AR symptoms on daily activities has previously been shown to be greater for AR patients than those with diabetes mellitus or hypertension.[20] Others have shown that living with symptomatic AR can impair school performance,[8] impair driving ability to the same extent as a blood alcohol level of 0.05%,[21] reduce work productivity[10 22] and induce absenteeism.[10] The latter two carry a hefty price tag, with absenteeism due to AR estimated to cost the UK economy £1.14 billion/year[10] and the cost of lost productivity due to AR thought to exceed that of chronic heart disease, asthma, diabetes, hypertension and respiratory infections combined![22]

For peer review only - http://bmjopen.bmj.com/site/about/guidelines.xhtml

The results of this study also show that the most commonly prescribed AR treatments in the UK do not provide adequate symptom relief for many patients, further endorsing the need for MP-AzeFlu. Prescribing physicians considered that other therapies were not sufficient in the past for 66.4% of patients and would not provide adequate symptom relief for 34.5% of patients. Most patients prescribed MP-AzeFlu had previously been treated with INS and/or oral anti-histamines. Inadequacy of previous AR treatments was also evident from the high symptomatic burden on treatment reported by patients, the high degree of polypharmacy noted (79.3%) and the high incidence of use of systemic corticosteroids (18.7%) and decongestants (45.6%). The former are rarely indicated for the management of rhinitis (except for severe obstruction or short-term rescue use),[23] whilst long-term use of the latter has been associated with rhinitis medicamentosa. A previous survey conducted in the UK also revealed that patients retained a high nasal and ocular symptom burden despite treatment with mono- or multiple AR therapies.[10] Furthermore, a retrospective observational study with data from the Optimum Patient Care Research Database showed that in the UK monotherapy with antihistamines or INS provided insufficient symptom relief for about 20% of SAR patients.[24] AR monotherapy was prescribed first for most patients, but by season end 45% of SAR patients had received multiple therapy, a practice which is not recommended by ARIA[19] due to insufficient evidence.[25 26] The current British Society for Allergy & Clinical Immunology (BSACI) UK guidelines suggest that additional therapies should be considered for brief targeted symptom relief only.[23]

The concept of achieving symptom relief (i.e. reduction in symptom severity) has been now been superseded by the concept of achieving symptom control.[14] A simple visual analogue

For peer review only - http://bmjopen.bmj.com/site/about/guidelines.xhtml

scale (VAS) is recommended by MACVIA ARIA as the new language of AR control.[12] The VAS has been used extensively in the field of AR to assess symptom severity[27] and treatment effectiveness[28], to define phenotypes, such as severe chronic upper airway disease,[29] and to define clinically relevant effect.[30] A VAS score of 5/10 cm is the cut-off used to define control and guide treatment decisions as part of an updated ARIA AR treatment algorithm called the AR CDSS.[14] It has also been incorporated into an app (*Allergy* Diary) to empower patients to take control of their own AR.[12] Data obtained from several countries (Germany, Sweden, Norway, Denmark and Romania) which gathered real-life data as part of a multi-national pan-European study have shown that on average patients treated with MP-AzeFlu achieve this AR CDSS VAS score cut-off (i.e. 5 cm) prior to Day 3, with 1 in 2 patients perceiving their AR as well-controlled at that time point.[31-35] The UK data presented here are taken from the same multi-national pan-European study. Although, effectiveness data were not recorded in the UK arm, results are expected to be consistent with those found in other countries with similar patient characteristics. MP-AzeFlu has the potential to reduce costs associated with managing AR in the UK, by reducing multiple prescriptions, number of doctor visits, referrals to secondary care and potentially reducing the number of patients referred for allergen-specific immunotherapy (AIT); supporting UK NHS cost-saving initiatives to manage more chronic conditions in the primary care setting.

The limitations of this study are those typically associated with non-interventional, observational datasets as the standard methods of blinding and randomisation cannot be applied. Thus, confounding can never safely be ruled out. Another limitation is that our study included no investigation of the effectiveness of MP-AzeFlu, and there was no direct

For peer review only

For peer review only - http://bmjopen.bmj.com/site/about/guidelines.xhtml

head to head comparison of MP-AzeFlu with other AR treatments. However, the effectiveness of MP-AzeFlu has been demonstrated in real-life studies conducted in other countries.[17] The strength of the evidence is derived from the inclusion of a sufficiently large patient population to draw general conclusion on use and safety of MP-AzeFlu in routine clinical practice in UK. The type of patient prescribed MP-AzeFlu in the UK was consistent with the patient profile identified in other countries including Germany, Sweden, Denmark and Romania, supporting the generalizability of our data to a wider pan-European population. In the pooled analysis for all countries as well as for the presented UK data the study data were complete for most demographic variables, therefore missing data are not considered as a potential source of bias for the study results.

In conclusion, the symptomatic burden of AR remains high for many patients in the UK, who live with uncontrolled disease, despite treatment with mono- and multiple therapies and repeat doctor visits. In the UK, MP-AzeFlu was prescribed for individuals (≥12 years) with moderate/severe AR irrespective of (i) previous AR treatment (mono or multiple), (ii) previous or likely treatment failure, (iii) phenotype, (iv) number of previous physician visits for AR and (v) for the relief of both acute symptoms and in anticipation of allergen exposure. A more effective treatment option, like MP-AzeFlu, with established superiority over intranasal corticosteroid and anti-histamine monotherapy, should improve AR control.

For peer review only - http://bmjopen.bmj.com/site/about/guidelines.xhtml

ACKNOWLEDGEMENTS

The authors thank the following investigators for their participation in the study: Mr Carl Philpott, James Paget Hospital, Great Yarmouth; Dr Dermot Dalton North Devon District Hospital, Barnstable; Dr Sadie Khawaja, Stepping Hill Hospital, Stockport; Dr Tom Dawson, Worcester Royal Hospital, West Midlands; Mr Alagar Chandra-Mohan, Ysbyty Gwynedd Hospital, Wrexham, Wales; Mr Arul Puveendran, Sunderland Royal Hospital, Sunderland; Mr Paul Counter, Cumberland Infirmary, Carlisle; Mr Ramsam Ullah, Royal Victoria Hospital, Belfast, N.Ireland; Mr San Sunkaraneni, Royal Surrey Hospital, Guildford; Prof Chris Raine, Bradford Royal Infirmary, Bradford. We also gratefully acknowledge each of the clinical research facilities which took part in this study.

Medical writing assistance in the preparation of this manuscript was provided by Dr Ruth Murray and Dr Winnie McFadzaen, Medscript Ltd, funded by MEDA Pharma GmbH & Co. KG. We thank Dr DunTung Nguyen and Dr Hans-Christian Kuhl for critical assessment of the data and manuscript review. This work was supported by MEDA Pharma GmbH & Co. KG.

Contribution of authors

All authors meet the authorship standards as outlined by the International Committee of Medical Journal Editors. They have all substantially contributed to the analysis and interpretation of data contained in this study, have been integrally involved in drafting and revising the article and have provided final approval for publication. All authors have agreed to be accountable for all aspects of the work in ensuring that questions related to the accuracy or integrity of any part of this work are appropriately investigated and resolved.

For peer review only

For peer review only - http://bmjopen.bmj.com/site/about/guidelines.xhtml

COMPETING INTERESTS

GS has received fees for advising Meda and for speaking at meetings on Dymista.

DP has received fees for advising Meda, research grants from Meda for studies on allergic rhinitis and fees for speaking at meetings and symposia supported by Meda

TE has received educational support, speaker fees and/or advisory board fees from Meda, Allergy Therapeutics and ALK.

SKA has received fees for speaking at a meeting by Meda.

JR has received fees for speaking at a meeting by Meda.

RS has received honorarium in MEDA sponsored educational events.

NK has received fees for advising and for speaking at meetings arranged by Meda

For peer review only - http://bmjopen.bmj.com/site/about/guidelines.xhtml

REFERENCES

1. Bauchau V, Durham SR. Prevalence and rate of diagnosis of allergic rhinitis in Europe. *Eur Respir J* 2004;24(5):758-64. doi: 10.1183/09031936.04.00013904

2. Bauchau V, Durham SR. Epidemiological characterization of the intermittent and persistent types of allergic rhinitis. *Allergy* 2005;60(3):350-3. doi: 10.1111/j.1398-9995.2005.00751.x

3. Scadding GK, Williams A. The burden of allergic rhinitis as reported by UK patients compared with their doctors. *Rhinology* 2008;46(2):99-106.

4. Meltzer EO. Allergic rhinitis: the impact of discordant perspectives of patient and physician on treatment decisions. *Clin Ther* 2007;29(7):1428-40. doi: 10.1016/j.clinthera.2007.07.013

5. Valovirta E, Myrseth SE, Palkonen S. The voice of the patients: allergic rhinitis is not a trivial disease. *Curr Opin Allergy Clin Immunol* 2008;8(1):1-9. doi: 10.1097/ACI.0b013e3282f3f42f

6. Bousquet PJ, Demoly P, Devillier P, et al. Impact of allergic rhinitis symptoms on quality of life in primary care. *Int Arch Allergy Immunol* 2013;160(4):393-400. doi: 10.1159/000342991

7. Scadding GK, Richards DH, Price MJ. Patient and physician perspectives on the impact and management of perennial and seasonal allergic rhinitis. *Clin Otolaryngol Allied Sci* 2000;25(6):551-7.

8. Walker S, Khan-Wasti S, Fletcher M, et al. Seasonal allergic rhinitis is associated with a detrimental effect on examination performance in United Kingdom teenagers: case-

For peer review only - http://bmjopen.bmj.com/site/about/guidelines.xhtml

control study. *J Allergy Clin Immunol* 2007;120(2):381-7. doi: 10.1016/j.jaci.2007.03.034

9. Clatworthy J, Price D, Ryan D, et al. The value of self-report assessment of adherence, rhinitis and smoking in relation to asthma control. *Prim Care Respir J* 2009;18(4):300-5. doi: 10.4104/pcrj.2009.00037

10. Price D, Scadding G, Ryan D, et al. The hidden burden of adult allergic rhinitis: UK healthcare resource utilisation survey. *Clin Transl Allergy* 2015;5:39. doi: 10.1186/s13601-015-0083-6

11. European Academy of Allergy and Clinical Immunology (EAACI). Beware of Allergy Campaign [Available from: http://www.bewareofallergy.com accessed July 2016.

12. Bousquet J, Schunemann HJ, Fonseca J, et al. MACVIA-ARIA Sentinel NetworK for allergic rhinitis (MASK-rhinitis): the new generation guideline implementation. *Allergy* 2015;70(11):1372-92. doi: 10.1111/all.12686

13. European Innovation Partnership on A, Healthy Ageing APB, Mechanisms of the Development of Allergy WP, et al. Integrated care pathways for airway diseases (AIRWAYS-ICPs). *Eur Respir J* 2014;44(2):304-23. doi: 10.1183/09031936.00014614

14. Bousquet J, Schunemann H, Arnavielhe S, et al. MACVIA clinical decision algorithm in allergic rhinitis in adolescents and adults. *J Allergy Clin Immunol* 2016;138:367-74.

15. Dymista. Summary of Product Characteristics [Available from: https://www.medicines.org.uk/emc/medicine/27579.

16. Meltzer E, Ratner P, Bachert C, et al. Clinically relevant effect of a new intranasal therapy (MP29-02) in allergic rhinitis assessed by responder analysis. *Int Arch Allergy Immunol* 2013;161(4):369-77. doi: 10.1159/000351404

For peer review only - http://bmjopen.bmj.com/site/about/guidelines.xhtml

For peer review only

17. Klimek L, Bachert, C., Stjarne, P., Dollner, R., Larsen, P., Haahr, P., Agache, I., Scadding, G., Price, D. MP-AzeFlu provides rapid and effective allergic rhinitis control in real-life: a pan-European study. *Allergy Asthma Proc* 2016;37(5):376.

18. Price D, Smith P, Hellings P, et al. Current controversies and challenges in allergic rhinitis management. *Expert Rev Clin Immunol* 2015;11(11):1205-17. doi: 10.1586/1744666X.2015.1081814

19. Bousquet J, Khaltaev N, Cruz AA, et al. Allergic Rhinitis and its Impact on Asthma (ARIA) 2008 update (in collaboration with the World Health Organization, GA(2)LEN and AllerGen). *Allergy* 2008;63 Suppl 86:8-160. doi: 10.1111/j.1398-9995.2007.01620.x

20. de la Hoz Caballer B, Rodriguez M, Fraj J, et al. Allergic rhinitis and its impact on work productivity in primary care practice and a comparison with other common diseases: the Cross-sectional study to evAluate work Productivity in allergic Rhinitis compared with other common dIseases (CAPRI) study. *Am J Rhinol Allergy* 2012;26(5):390-4. doi: 10.2500/ajra.2012.26.3799

21. Vuurman EF, Vuurman LL, Lutgens I, et al. Allergic rhinitis is a risk factor for traffic safety. *Allergy* 2014;69(7):906-12. doi: 10.1111/all.12418

22. Lamb CE, Ratner PH, Johnson CE, et al. Economic impact of workplace productivity losses due to allergic rhinitis compared with select medical conditions in the United States from an employer perspective. *Curr Med Res Opin* 2006;22(6):1203-10. doi: 10.1185/030079906X112552

23. Scadding GK, Durham SR, Mirakian R, et al. BSACI guidelines for the management of allergic and non-allergic rhinitis. *Clin Exp Allergy* 2008;38(1):19-42. doi: 10.1111/j.1365-2222.2007.02888.x

For peer review only - http://bmjopen.bmj.com/site/about/guidelines.xhtml

24. Price DB, Scadding G, Bachert C, et al. UK prescribing practices as proxy markers of unmet need in allergic rhinitis: a retrospective observational study. *NPJ Prim Care Respir Med* 2016;26:16033. doi: 10.1038/npjpcrm.2016.33

25. Anolik R, Mometasone Furoate Nasal Spray With Loratadine Study G. Clinical benefits of combination treatment with mometasone furoate nasal spray and loratadine vs monotherapy with mometasone furoate in the treatment of seasonal allergic rhinitis. *Ann Allergy Asthma Immunol* 2008;100(3):264-71. doi: 10.1016/S1081-1206(10)60452-8

26. Esteitie R, deTineo M, Naclerio RM, et al. Effect of the addition of montelukast to fluticasone propionate for the treatment of perennial allergic rhinitis. *Ann Allergy Asthma Immunol* 2010;105(2):155-61. doi: 10.1016/j.anai.2010.05.017

27. Bousquet PJ, Combescure C, Neukirch F, et al. Visual analog scales can assess the severity of rhinitis graded according to ARIA guidelines. *Allergy* 2007;62(4):367-72. doi: 10.1111/j.1398-9995.2006.01276.x

28. Bousquet J, Bachert C, Canonica GW, et al. Efficacy of desloratadine in intermittent allergic rhinitis: a GA(2)LEN study. *Allergy* 2009;64(10):1516-23. doi: 10.1111/j.1398-9995.2009.02115.x

29. Bousquet PJ, Bachert C, Canonica GW, et al. Uncontrolled allergic rhinitis during treatment and its impact on quality of life: a cluster randomized trial. *J Allergy Clin Immunol* 2010;126(3):666-8 e1-5. doi: 10.1016/j.jaci.2010.06.034

30. Demoly P, Bousquet PJ, Mesbah K, et al. Visual analogue scale in patients treated for allergic rhinitis: an observational prospective study in primary care: asthma and rhinitis. *Clin Exp Allergy* 2013;43(8):881-8. doi: 10.1111/cea.12121

For peer review only - http://bmjopen.bmj.com/site/about/guidelines.xhtml

31. Klimek L, Bachert C, Mosges R, et al. Effectiveness of MP29-02 for the treatment of allergic rhinitis in real-life: results from a noninterventional study. *Allergy Asthma Proc* 2015;36(1):40-7. doi: 10.2500/aap.2015.36.3823

32. Stjarne P, Strand V, Theman K, et al. Real-life effectiveness of a new allergic rhinitis thrapy (MP29-02) in Sweden. *Allergy* 2015;70(504)

33. Dollner R, Lorentz Larsen P, Sheyauldeen S, et al. Real-life effectiveness of a new allergic rhinitis therapy (MP29-02) in Norway. *Allergy* 2015;70(502)

34. Haahr P, Jacobsen C, Blegvad S, et al. Real life effectiveness of a new allergic rhinitis therapy (MP29-02) in Denmark. *Allergy* 2015;70(500)

35. Agache ISCL, P; Bucur, I; Doros, I-C; Poenaru, M. Real-life effectiveness of a new allergic rhinitis therapy (MP-AzeFlu*) in Romania. *Allergy* 2016;71(Suppl S102):386.

For peer review only

For peer review only - http://bmjopen.bmj.com/site/about/guidelines.xhtml

LEGEND TO FIGURES

Figure 1

Number of previous physician visits for allergic rhinitis (AR) in the current calendar year prior to prescribing MP-AzeFlu. N=193 patients with moderate-to-severe AR attending routine clinical practice in the UK.

Figure 2

Most frequent reasons reported by physicians for prescribing MP-AzeFlu to patients with moderate-to-severe allergic rhinitis (n=193) attending routine clinical practice in the UK. Tx: treatment

Figure 3

Number of allergic rhinitis (AR) treatments (prescription and over the counter) used in the last calendar year by AR patients with moderate-to-severe AR (n=193) attending routine clinical practice in the UK

For peer review only - http://bmjopen.bmj.com/site/about/guidelines.xhtml

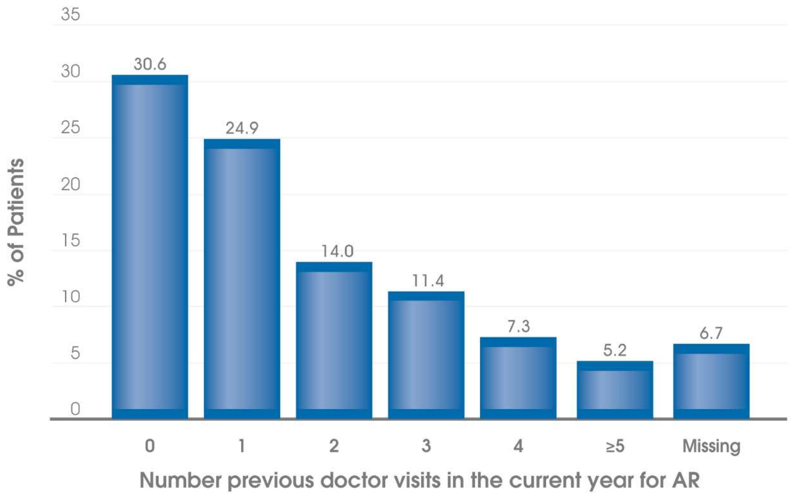

Number of previous physician visits for allergic rhinitis (AR) in the current calendar year prior to prescribing MP-AzeFlu. N=193 patients with moderate-to-severe AR attending routine clinical practice in the UK.

200x100mm (300 x 300 DPI)

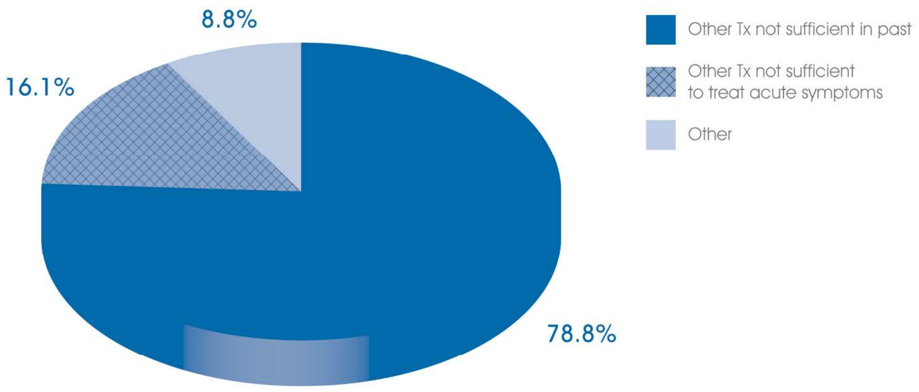

Most frequent reasons reported by physicians for prescribing MP-AzeFlu to patients with moderate-to-severe allergic rhinitis (n=193) attending routine clinical practice in the UK. Tx: treatment

200x100mm (300 x 300 DPI)

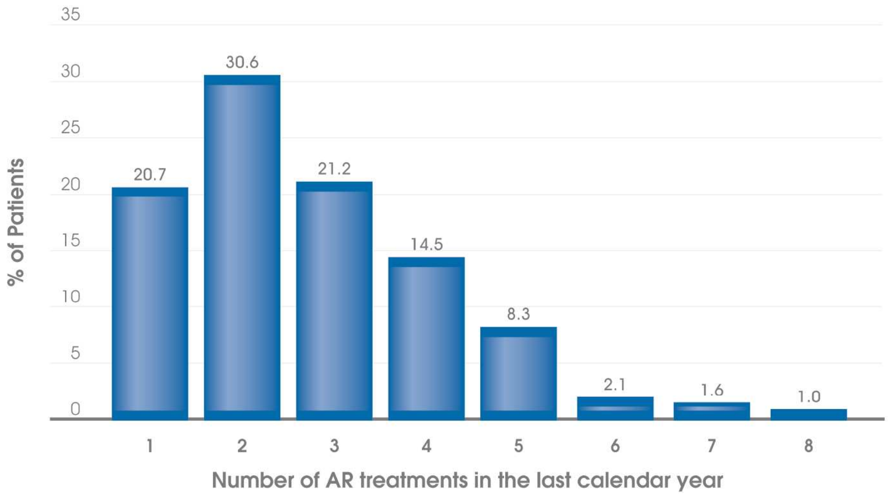

Number of allergic rhinitis (AR) treatments (prescription and over the counter) used in the last calendar year by AR patients with moderate-to-severe AR (n=193) attending routine clinical practice in the UK

200x100mm (300 x 300 DPI)

review only

STROBE Statement—checklist of items that should be included in reports of observational studies

	Item No	Recommendation		Page
**Title and abstract**	1	(*a*) Indicate the study's design with a commonly used term in the title or the abstract	X	1
		(*b*) Provide in the abstract an informative and balanced summary of what was done and what was found	X	2-3
**Introduction**				
Background/rationale	2	Explain the scientific background and rationale for the investigation being reported	X	5-6
Objectives	3	State specific objectives, including any prespecified hypotheses	X	6
**Methods**				
Study design	4	Present key elements of study design early in the paper	X	7
Setting	5	Describe the setting, locations, and relevant dates, including periods of recruitment, exposure, follow-up, and data collection	X	7-8
Participants	6	(*a*) *Cohort study*—Give the eligibility criteria, and the sources and methods of selection of participants. Describe methods of follow-up<br>*Case-control study*—Give the eligibility criteria, and the sources and methods of case ascertainment and control selection. Give the rationale for the choice of cases and controls<br>*Cross-sectional study*—Give the eligibility criteria, and the sources and methods of selection of participants	X	7-8
		(*b*) *Cohort study*—For matched studies, give matching criteria and number of exposed and unexposed<br>*Case-control study*—For matched studies, give matching criteria and the number of controls per case	NA	
Variables	7	Clearly define all outcomes, exposures, predictors, potential confounders, and effect modifiers. Give diagnostic criteria, if applicable	X	8-9
Data sources/ measurement	8*	For each variable of interest, give sources of data and details of methods of assessment (measurement). Describe comparability of assessment methods if there is more than one group	*X*	*9*
Bias	9	Describe any efforts to address potential sources of bias	X	3
Study size	10	Explain how the study size was arrived at	X	9
Quantitative variables	11	Explain how quantitative variables were handled in the analyses. If applicable, describe which groupings were chosen and why	NA	
Statistical methods	12	(*a*) Describe all statistical methods, including those used to control for confounding	X	9
		(*b*) Describe any methods used to examine subgroups and interactions	NA	
		(*c*) Explain how missing data were addressed	X	19
		(*d*) *Cohort study*—If applicable, explain how loss to follow-up was addressed<br>*Case-control study*—If applicable, explain how matching of cases and controls was addressed<br>*Cross-sectional study*—If applicable, describe analytical methods taking account of sampling strategy	NA	
		(*e*) Describe any sensitivity analyses		

Continued on next page

For peer review only - http://bmjopen.bmj.com/site/about/guidelines.xhtml

**Results**				
Participants	13*	(a) Report numbers of individuals at each stage of study—eg numbers potentially eligible, examined for eligibility, confirmed eligible, included in the study, completing follow-up, and analysed	X	10
		(b) Give reasons for non-participation at each stage	X	10
		(c) Consider use of a flow diagram	NA	
Descriptive data	14*	(a) Give characteristics of study participants (eg demographic, clinical, social) and information on exposures and potential confounders	X	10-12
		(b) Indicate number of participants with missing data for each variable of interest	NA	
		(c) *Cohort study*—Summarise follow-up time (eg, average and total amount)	NA	
Outcome data	15*	*Cohort study*—Report numbers of outcome events or summary measures over time	X	*12-14*
		Case-control study—Report numbers in each exposure category, or summary measures of exposure	NA	
		Cross-sectional study—Report numbers of outcome events or summary measures	NA	
Main results	16	(*a*) Give unadjusted estimates and, if applicable, confounder-adjusted estimates and their precision (eg, 95% confidence interval). Make clear which confounders were adjusted for and why they were included	NA	
		(*b*) Report category boundaries when continuous variables were categorized	NA	
		(*c*) If relevant, consider translating estimates of relative risk into absolute risk for a meaningful time period	NA	
Other analyses	17	Report other analyses done—eg analyses of subgroups and interactions, and sensitivity analyses	NA	
**Discussion**				
Key results	18	Summarise key results with reference to study objectives	X	15 & 19
Limitations	19	Discuss limitations of the study, taking into account sources of potential bias or imprecision. Discuss both direction and magnitude of any potential bias	X	18
Interpretation	20	Give a cautious overall interpretation of results considering objectives, limitations, multiplicity of analyses, results from similar studies, and other relevant evidence	X	19
Generalisability	21	Discuss the generalisability (external validity) of the study results	X	19
**Other information**				
Funding	22	Give the source of funding and the role of the funders for the present study and, if applicable, for the original study on which the present article is based		20

*Give information separately for cases and controls in case-control studies and, if applicable, for exposed and unexposed groups in cohort and cross-sectional studies.

Note: An Explanation and Elaboration article discusses each checklist item and gives methodological background and published examples of transparent reporting. The STROBE checklist is best used in conjunction with this article (freely available on the Web sites of PLoS Medicine at http://www.plosmedicine.org/, Annals of Internal Medicine at http://www.annals.org/, and Epidemiology at http://www.epidem.com/). Information on the STROBE Initiative is available at www.strobe-statement.org.

For peer review only
